# Genome-Wide Identification, Phylogeny, Evolution and Expression Patterns of AP2/ERF Genes and Cytokinin Response Factors in *Brassica rapa* ssp. *pekinensis*

**DOI:** 10.1371/journal.pone.0083444

**Published:** 2013-12-30

**Authors:** Zhenning Liu, Lijun Kong, Mei Zhang, Yanxia Lv, Yapei Liu, Minghau Zou, Gang Lu, Jiashu Cao, Xiaolin Yu

**Affiliations:** 1 Laboratory of Cell & Molecular Biology, Institute of Vegetable Science, Zhejiang University, Hangzhou, People’s Republic of China; 2 Laboratory of Horticultural Plant Growth & Quality Regulation, Ministry of Agriculture, Hangzhou, People’s Republic of China; University of New England, Australia

## Abstract

The AP2/ERF transcription factor family is one of the largest families involved in growth and development, hormone responses, and biotic or abiotic stress responses in plants. In this study, 281 AP2/ERF transcription factor unigenes were identified in Chinese cabbage. These superfamily members were classified into three families (AP2, ERF, and RAV). The ERF family was subdivided into the DREB subfamily and the ERF subfamily with 13 groups (I– XI) based on sequence similarity. Duplication, evolution and divergence of the AP2/ERF genes in *B. rapa* and *Arabidopsis thaliana* were investigated and estimated. Cytokinin response factors (CRFs), as a subclade of the AP2/ERF family, are important transcription factors that define a branch point in the cytokinin two-component signal (TCS) transduction pathway. Up to 21 *CRFs* with a conserved CRF domain were retrieved and designated as *BrCRFs*. The amino acid sequences, conserved regions and motifs, phylogenetic relationships, and promoter regions of the 21 *BrCRFs* were analyzed in detail. The *BrCRFs* broadly expressed in various tissues and organs. The transcripts of *BrCRFs* were regulated by factors such as drought, high salinity, and exogenous 6-BA, NAA, and ABA, suggesting their involvement in abiotic stress conditions and regulatory mechanisms of plant hormone homeostasis. These results provide new insight into the divergence, variation, and evolution of AP2/ERF genes at the genome-level in Chinese cabbage.

## Introduction

Abiotic stress conditions such as drought and high salinity are the most common stress factors that adversely affect plant growth and yield. Plants have evolved a complex signaling network at the molecular, cellular, and system levels to survive and flourish in varied environments [1]. Many aspects of adaptation, including developmental, physiologic, and biochemical changes, are regulated by stress responsive gene expression. Transcription factors (TF) play pivotal functions in signal transduction to activate or suppress defense response genes and regulate the interactions between different signaling pathways. More than 1500 genes encode TFs in *Arabidopsis*, accounting for more than 7% of the protein coding genes [2]–[4]. The AP2/ERF superfamily, one of the largest groups of TFs in plants, is characterized by the presence of AP2/ERF-type DNA-binding domains that consist of 60–70 highly conserved amino acids and plays significant roles in regulating abiotic stress-responsive genes expression in plants [5], [6]. AP2/ERF TFs are involved in plant growth and development [7]–[11], hormone response [7], [12]–[15], and biotic or abiotic stress responses [16]–[18]. AP2/ERF TFs have been identified in various plant species, including Arabidopsis [5], [19], rice [19]–[21], maize [22], soybean [23], tomato [1], cucumber [24], Chinese cabbage [25]–[27], poplar [28], grape [29], and plum [30], among others. Arabidopsis AP2/ERF TF genes are classified into three groups based on the number and similarity of their DNA-binding domains: AP2, RAV, and ERF families. The ERF family could be divided into two major subfamilies: the dehydration-responsive element-binding protein (DREB) subfamily and ethylene responsive factor (ERF) subfamily, which are further divided into groups I to X [5], [19]. Several closely related members of the Arabidopsis AP2/ERF gene family that are upregulated by cytokinin are identified and designated as cytokinin response factors (*CRFs*) [7]. CRFs are AP2/ERF TFs that occur as the B-5 or VI and B-6 or VI-L phylogenetic clades of AP2/ERF proteins and contain a single AP2 DNA-binding domain [7], [19], [31]. Aside from the conserved AP2 domain, CRF proteins have a representative CRF domain in the N-terminal region, a novel domain of approximately 65 amino acids. CRF domain-containing proteins are found in liverworts, mosses, lycopods, ferns, conifers, and all major lineages of flowering plants [31]. The expression of some *CRFs* could be regulated by abiotic stress and various plant hormones. CRF proteins also appear to form a branch of the cytokinin signaling pathway and may independently regulate downstream cytokinin targets or in conjunction with type-B response regulators [7], [32]. Previous research showed that CRF domain proteins alone could form both homodimers and heterodimers with each other and specifically interact directly with most Arabidopsis histidine phosphotransfer proteins (AHP1–AHP5). This is the first described ability of the CRF domain in plants. *CRFs* are involved in plant growth and development and participate in stress tolerance networks. Analyzing loss-of-function mutations revealed that *CRFs* redundantly regulate the development of embryos, cotyledons, and leaves [7]. Transgenic Arabidopsis, which constitutively overexpress *CRF2*, exhibit more and smaller chloroplasts per cell than wild-type plants [9]. Microarray expression studies revealed that cold stress induces *CRF2* transcription, whereas *CRF5* transcription in the roots is strongly induced during salt stress [33]. However, only 12 *AtCRF* and 11 *SlCRF* genes were identified and characterized in detail. Information on the *CRF* genes in other species remains limited and their biological functions still need further research.

*Brassica* crops are used for human nutrition and are important in daily life. Chinese cabbage (*B. rapa* ssp. *pekinensis*) is one of the most important *B. rapa* crops and is an economically important vegetable worldwide because of its high yield and good quality. Whole genome sequencing of *B. rapa* (Chiifu-401-42) by The *Brassica rapa* Genome Sequencing Project Consortium [34] enables the genome-wide identification and functional study of gene families related to the morphologic diversity and agronomic traits of *Brassica* crops [35]. The ‘A’ genome of *B. rapa* is an important resource for studying the evolution of polyploidy genomes and potential strategies for genetically improving *Brassica*-related crops [26]. *CRFs* play vital regulatory roles in various developmental processes and stimuli responses in plants. Therefore, comprehensively analyzing the phylogenetic relationships, conserved motifs, and differential expression patterns across plant tissues and response mechanisms to various stress conditions and plant hormones is crucial for studying the physiologic functions of these genes to improve yield and making the crop better suited to diverse environmental conditions.

Previous studies reported AP2/ERF family TFs in Chinese cabbage. The expression patterns of several AP2/ERF genes under cold and heat stress were examined [25], [26], [36]. However, AP2/ERF family TFs are involved in various abiotic and biotic stresses, not merely adverse temperature conditions. Moreover, information on the *CRFs* characterized by a specific CRF domain in Chinese cabbage remains lacking. The *Brassica* Database was surveyed to gain further information on the AP2/ERF superfamily and its subclade *CRFs* in Chinese cabbage. A total of 281 members were identified in this superfamily, including 131 ERF genes, 105 DREB genes, 30 AP2 genes, 14 RAV genes, and 1 soloist. The ERF family was subdivided into 13 groups (I– XI), with 21 *BrCRFs* classified in groups VI and VI-L. Their structure and phylogeny were comprehensively analyzed. The expression patterns of the 21 *BrCRFs* were characterized in detail. The results from this study will serve as a basis for the functional analyses of AP2/ERF genes, especially the *CRF* genes in Chinese cabbage.

## Materials and Methods

### Identification of AP2/ERF and CRF gene families in the *B. rapa* genome

The conserved AP2 and CRF domains of Arabidopsis AP2/ERF and CRF protein sequences were originally applied as seed sequences to search the *Brassica* Database Version 1.1 (http://brassicadb.org/brad/) [34], [37], [38] and the NCBI database (www.ncbi.nlm.nih.gov). The search was based on a BLASTP search with an expected value of 100. The identified CRFs were used as queries to reconfirm the multiple databases to ensure that no additional related genes were missing from the database. All of the sequences that met the requirements were analyzed to eliminate genes that did not contain the known conserved domains and motifs using the Pfam database (http://pfam.janelia.org/) [39], the SMART database (http://smart.embl-heidelberg.de/) [40], and Conserved Domain Database of the NCBI (http://www.ncbi.nlm.nih.gov/Structure/cdd/wrpsb.cgi) [41]. Also CRFs in the poplar, moss, and alga were searched using the Phytozome v9.0 GBrowse database (http://www.phytozome.net/) [42].

### Motif recognition, multiple-sequence alignment, and phylogenetic analysis

The online MEME (http://meme.sdsc.edu/meme/meme.html) was used to identify the *Br*CRF motifs with expected e-values less than 2×10^−30^ [43], [44]. The alignment of the identified AP2/ERF protein sequences was performed with a gap open penalty of 10 and gap extension penalty of 0.2 using ClustalW implemented in MEGA5.0 software (http://www.megasoftware.net/) [45]. Unrooted phylogenetic tree was constructed using the neighbor-joining (NJ) method, with Poission correction, pairwise deletion and bootstrap (1,000 replicates; random seeds), as parameters. For the phylogenetic tree of CRF proteins from *Arabidopsis thaliana*, *Brassica rapa*, *Oryza sativa*, *Zea mays*, *Solanum lycopersicum*, *Populus trichocarpa*, and *Physcomitrella patens*, the complete CRF protein sequences were used with methods previously described.

### Composition and physical/chemical characterization analysis

The gene structure schematic of *BrCRFs* was drawn using the Gene Structure Display Server (http://gsds.cbi.pku.edu.cn/). The model of the *Br*CRF protein forms, including size, domains, and motifs, were drawn artificially. The number and percentage of Ser in the deduced amino acid sequences were calculated. The isoelectric point (pI) of the *Br*CRFs were predicted using the Compute pI/Mw software (http://www.expasy.ch/tools/pi_tool.html) [46]. The subcellular localization was predicted using PSORT (http://psort.hgc.jp/) [47].

### Analysis of the putative promoter regions of *BrCRF* genes

2,000-bp upstream sequences of the transcriptional start site of each *BrCRFs* were chosen to identify the *cis*-elements in the putative promoter regions of the *BrCRFs*. The PLACE website (http://www.dna.affrc.go.jp/PLACE/) [48] was applied to identify the putative *cis*-regulatory elements along the promoter sequences. Motifs were analyzed using MEME for these 2,000-bp upstream sequences [44]. The gene ontology for the motifs were conducted through GOMO analysis [49].

### Chromosomal localization and gene duplications

The *Brassica* Genome Browse (BRAD; http://brassicadb.org/cgi-bin/gbrowse/cbgdb11/) was used to map the positions of the *AP2/ERF* TFs and *CRFs* of *B. rapa* to the physical maps of the ten *B. rapa* chromosomes. The distribution of *AP2/ERF* TFs of *A. thaliana* was visualized with Chromosome Map Tool (http://www.arabidopsis.org/jsp/ChromosomeMap/tool.jsp). Tandem duplications were defined if two genes were separated by four or fewer gene loci [50]. Segmental duplications were identified through synteny analysis using an online tool (http://chibba.agtec.uga.edu/duplication/) [51]. Duplications of *BrCRFs* were checked by searching homologous genes between Arabidopsis and three subgenomes (LF, MF1, and MF2) of *B. rapa* (http://brassicadb.org/brad/searchSynteny.php). Synteny of the *BrCRFs* were analyzed using the online PGDD (http://chibba.agtec.uga.edu/duplication/) [52]. The occurrence of duplication events and homologous genes divergence, as well as the selective pressure on duplicated genes, were estimated by calculating synonymous (*Ks*) and non-synonymous substitutions (*Ka*) per site between the duplicated gene pairs using the Codeml procedure of the PAML program [53]. The divergence time was calculated using the neutral substitution rate of 1.5×10^−8^ substitutions per site per year for the chalcone synthase gene (*Chs*) [54].

### Plant growth and treatments

The material (*B. rapa* ssp. *pekinensis* cv. Zhonghan No. 1), a widely cultivated variety in China, was grown at the experimental farm in Zhejiang University. Roots, floral stems, leaves, flowers, immature siliques, sepals, petals, stamens, pistils, little buds (<1.6 mm), middle buds (1.6 mm to 2.8 mm), and big buds (>2.8 mm) were sampled from at least ten plants to analyze tissue- and organ-specific expression. The diameter of the floral buds was measured using a vernier caliper.

*B. rapa* ssp. *pekinensis* line Chiifu-401-42 was used for treatment. All seedlings were grown under a 16 h light/8 h dark photoperiod at 25°C±1°C for about 3 weeks. Only the second true leaves were sampled to minimize differences. The nutrient solution was supplied with 200 mM NaCl for salt treatment. The leaves were separately collected at 0, 3, 8, and 16 h after stress induction. Three-week-old seedlings were withheld from watering to initiate the drought treatment. The leaves were divided into 4 levels based on the degree of drought: 0, I, II, III. The 0 represented that the leaves were normal with well-watered seedlings; I represented that the leaves started to wither; II represented that the leaves were severely withered; and III represented that the whole seedlings were withered. The 3-week-old seedlings were sprayed with 100 µM 6-BA for the cytokinin treatment, 100 µM NAA for the auxin treatment, and 100 µM ABA for the ABA treatment. The leaves were sampled at 0, 0.5, and 1 h after spraying, and the control was sprayed with double distilled water alone. All the materials sampled were immediately frozen in liquid nitrogen and stored in a refrigerator at -75°C.

### RNA extraction and qRT-PCR analysis

The total RNA was extracted using a TRIZOL reagent (Invitrogen, Germany) based on the manufacturer's instructions. The first cDNA strand was generated using the Takara Reverse Transcription system (Japan) following the manufacture's protocol. qRT-PCR was carried out as previously described [55] using the primers listed in Table S1. The primers were designed using the Primer 5.0 software. The specificity of each primer for its corresponding gene was checked using the BLASTN program of the BRAD. The specificity of the reactions was verified by melting curve analysis and products were further confirmed by agarose gel electrophoresis. Two biological replicates were performed with three technical replicates for each sample plus the negative control. The *BrActin1* gene was used as the reference gene. The comparative ΔΔ^CT^ method was used to calculate the relative expression levels of the different genes. The data of qRT-PCR were clustered using the average linkage method with Pearson correlation distance metric by Multiple Array Viewer [56].

## Results

### Identification, classification, and phylogenetic relationships of the AP2/ERF gene families in the *B. rapa* genome

**Figure 1:**
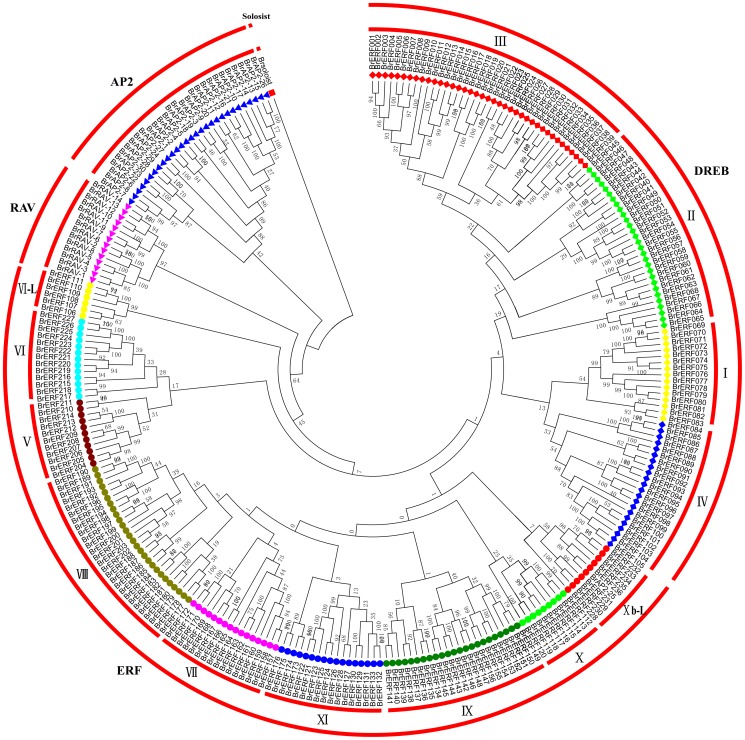
An unrooted phylogenetic tree of AP2/ERF family proteins in *B. rapa*. The complete sequences of 281 AP2/ERF family proteins identified in this study were aligned by ClustalW and the phylogenetic tree was constructed using the neighbor-joining method with MEGA5.0 software.

A total of 289 candidate genes with AP2 domains were retrieved from the *B. rapa* genome based on the BLASTP search against the *Brassica* Database Version 1.1(http://brassicadb.org/brad/) and the NCBI database, which is an equal number of Li's results [26]. Contradictorily, Song et al. recently identified 291 putative AP2/ERF transcription factor genes from the Chinese cabbage genome database [27]. In fact, locations of the two extra loci (*Bra023745* and *Bra027615*) were completely identical to their closest loci (*Bra023744* and *Bra027614*), thus they should not been considered as candidate genes. Besides, the sequences of *Bra027614* and *Bra023744* were identical to that of *Bra027616* and *Bra023746*, respectively. Therefore, the latter two genes were excluded for further study. A candidate gene with additional domains, except for the AP2 domain, was ruled out if the corresponding ortholog in Arabidopsis did not belong to the AP2/ERF superfamily, such as *Bra011244*, *Bra010258*, *Bra017612*, *Bra027447*, *Bra008793*, and *Bra023440*. A total of 281 genes in the *B. rapa* genome were finally identified as members of the AP2/ERF superfamily that encode AP2/ERF domain(s) (Table S2). An unrooted phylogenetic tree with *Br*AP2/ERF family proteins was constructed (Figure 1). A total of 25 genes were predicted to encode proteins with two complete or incomplete AP2/ERF domains and were assigned to the AP2 family. 5 genes were predicted to encode a complete or an incomplete AP2/ERF domain, whereas the AP2/ERF domains of the 5 genes were distinct from those of the members of the ERF family and were more closely related to those of the AP2 family. Thus, these genes were also assigned to the AP2 family. Up to 14 genes were predicted to encode one AP2/ERF domain and one B3 domain and were assigned to the RAV family. One gene, *Bra034895*, including an AP2-like domain sequence, had low homology with other AP2/ERF genes and was designated as a soloist. This soloist shared high similarity with *At4g13040*, another soloist in *A. thaliana* [19]. The remaining 236 genes assigned to the ERF family were further subclassified into two groups based on the similarity of the amino acid sequences of the AP2/ERF domains: 105 genes that encode a DREB-like protein were assigned to the DREB subfamily, and 131 genes that encode an ERF-like protein were assigned to ERF subfamily. The ERF family was subdivided into 13 groups, corresponding to groups I– XI in Arabidopsis and rice [19]. It was discordant that *Bra034249* encoded double AP2/ERF domains, but was clustered with group Xb-L in the ERF subfamily. It is possible that the gene was not full-length. Further analysis to obtain the full-length clone may group it into the AP2 subfamily. Therefore, *Bra034249* was still considered as a member of the AP2 subfamily.

The organization of the AP2/ERF superfamily genes in Chinese cabbage and the comparative distribution from Arabidopsis, rice, poplar, cucumber, and the Chinese plum were shown in Table 1. We performed a multiple sequence alignment using the complete amino acid sequences of the AP2/ERF proteins from each group to investigate the sequence features of the *Br*AP2/ERF family proteins, respectively (Figure S1A–D). All *Br*AP2/ERF family proteins were marked with one or two representative AP2/ERF domain(s). Groups VI and VI-L had an additional CRF domain with consensus core sequences [ATD×SS], which was the representative features of the CRF gene family members in a wide range of land plants [7], [31]. The total 21 genes in these two groups were designated as *BrCRFs* and were researched in detail. The members in Group VI had a mitogen-activated protein (MAP) kinase and/or casein kinase I functional site, whereas the members in Group VI-L possessed motifs with consensus sequences [FN×××L×IP] and [LPD×DF×D] [57]. The regions of the acidic amino acid–rich, Gln-rich, Pro-rich, and/or Ser/Thr-rich amino acid sequences are often designated as transcriptional activation domains [58]. Clusters of Ser-rich residues have been found in several AP2/ERF proteins and they may be involved in the activation of the transcription [59]–[61]. Ser rich regions were specifically recognized with members in Groups VIII and X. The number and percentage of Ser in each amino acid sequence of the deduced polypeptide were also calculated. We found 26 proteins whose Ser percent exceeded 15% (Table S2). Moreover, a total of 53 proteins were also found with at least 5 consecutive Ser (data not given). The Ser-rich region probably ensured the transcriptional activation of the AP2/ERF TFs in Chinese cabbage.

**Table 1:** Summary the AP2/ERF superfamily genes in *Brassica rapa*, compared with those in *Arabidopsis thaliana, Oryza sativa*, *Populus trichocarpa*, *Cucumis sativus*, and *Prunus mume*, as classified by Nakano et al., (2006).

Family	Subfamily	Group	*Brassica rapa*	*Arabidopsis thaliana*	*Oryza sativa*	*Populus trichocarpa*	*Cucumis sativus*	*Prunus mume*
**AP2**			**30**	**18**	**29**	**26**	**20**	**20**
**ERF**	**DREB**	Total	**105**	**57**	**56**	**66**	**42**	**35**
		I	15	10	9	5	5	5
		II	29	15	15	20	10	4
		III	39	23	26	35	20	20
		IV	22	9	6	6	7	6
	**ERF**	Total	**131**	**65**	**76**	**103**	**61**	**55**
		V	11	5	8	10	15	10
		VI	13	8	6	11	8	5
		VII	16	5	15	6	3	3
		VIII	27	15	13	17	11	10
		IX	23	17	18	42	16	18
		X	9	8	13	9	8	7
		VI -L	6	4	3	4	—	2
		Xb-L	9	3	—	4	—	0
	**A single group**		17	—	7	—	—	—
**RAV**			**14**	**6**	**5**	**6**	**4**	**5**
**Soloist**			**1**	**1**	**1**	**1**	**4**	**1**
**Total**			**281**	**147**	**174**	**202**	**131**	**116**

Totals for each family are in bold-type. — represents no genes identified in the group.

### Characterization of gene structure and deduced amino acid sequences of *BrCRFs*

**Figure 2:**
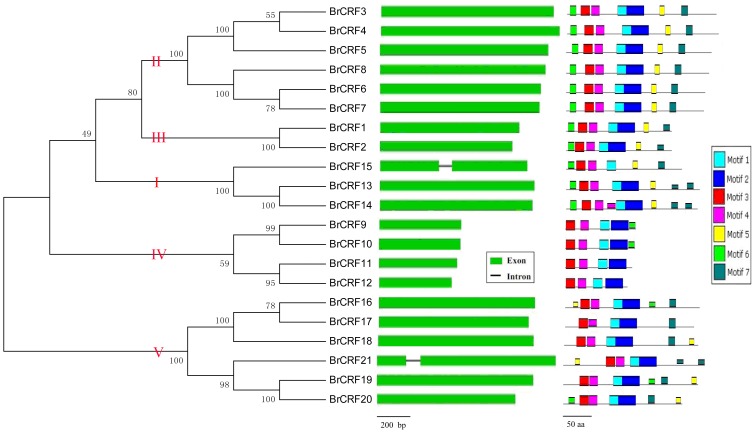
Phylogenetic relationships, gene structures and motifs analysis of the *BrCRFs*.

**Figure 3:**
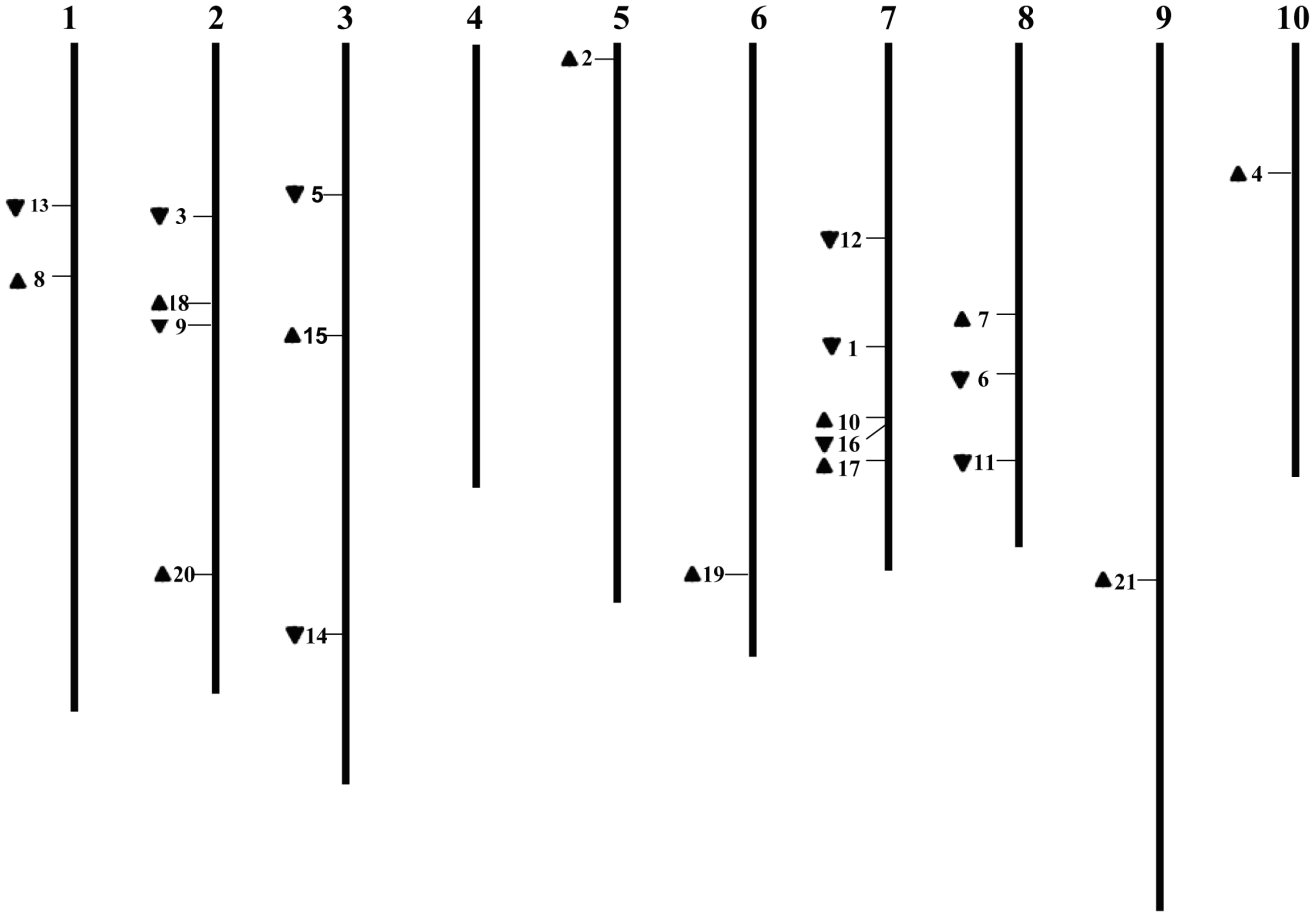
Chromosomal locations of *BrCRFs*. The arrows next to gene names show the direction of transcription.

**Figure 4:**
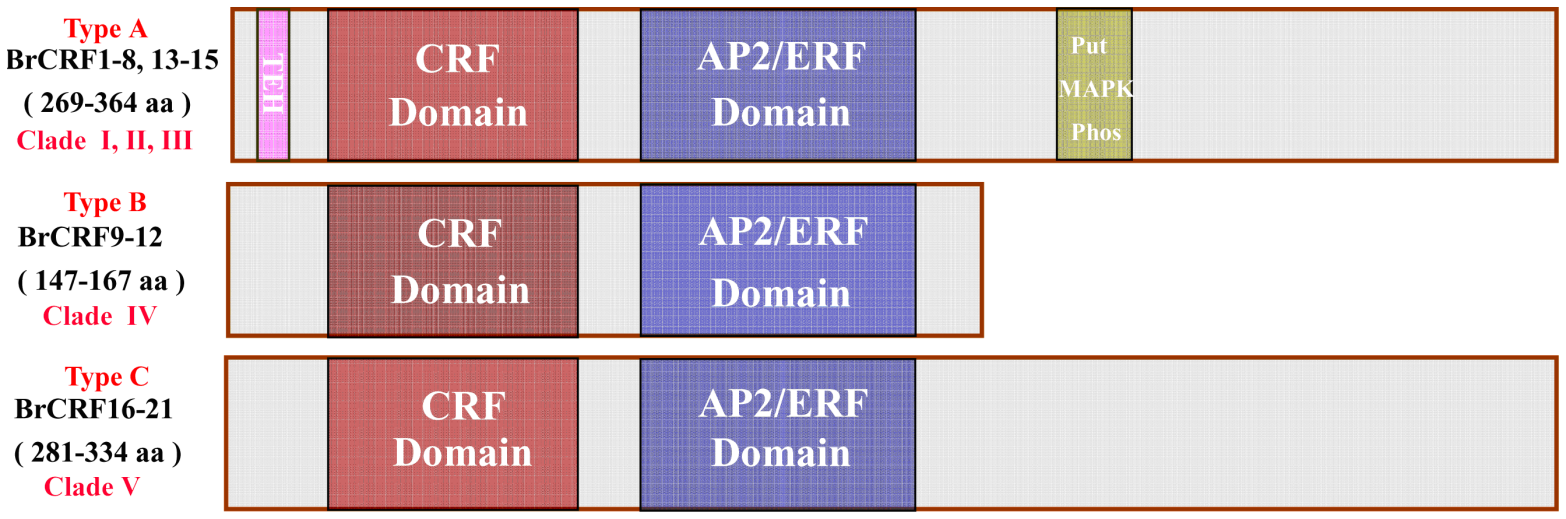
A model of *Br*CRF protein form including size, domains, and motifs for 21 *Br*CRFs.

Up to 21 *CRF* genes from Chinese cabbage, known as *BrCRFs*, were identified and characterized (Table 2). These genes are members of the AP2/ERF transcription factor family, specifically related to Group VI and VI-L of the ERF subfamily genes, known in Arabidopsis as *AtCRFs* [5], [19], [31]. To analyze the gene structural characteristics and deduced amino acid sequences of the *BrCRF* genes, protein sequences were aligned, phylogenetic relationships were analyzed, gene structures with exons and introns were made, and conserved regions and motifs were examined (Figure 2). As shown in Table 2 and Figure 3, the *BrCRFs* were distributed on 9 of 10 chromosomes (except for A04) belonging to 3 subgenomes (LF, MF1, and MF2). Consistent with *CRFs* in other plants, the ORF length of *BrCRFs* ranged from 444 bp to 1095 bp that encode 147 aa to 364 aa polypeptides, with predicted molecular weights varying from 16.1 kDa to 41.4 kDa. Based on the conserved regions and motifs analyses by MEME, *Br*CRFs at a protein level fell into three classifications, Type A, B, C (Figure 4). The Type A had 11 CRF proteins (*Br*CRF1–8 and *Br*CRF13–15) that contained both a specific CRF domain (Motif 3, 4) and an AP2 domain (Motif 1, 2) in addition to the TEH region (Motif 6) on the N-terminal region, and a putative mitogen-activated protein kinase (MAPK) phosphorylation site (Motif 5) on the C-terminal region, similar to that in Arabidopsis (*At*CRF1-6) [31]. The Type B included 3 shortened CRF proteins (*Br*CRF9-12) that contained a CRF and AP2 domain, but lacked the TEH region and the phosphorylation motif, as was also seen in Arabidopsis (*At*CRF7-8). Similar to *At*CRF9-12, the Type C was composed of 5 *Br*CRFs (*Br*CRF16-21), the length of deduced amino acid sequences was similar to the Type A, possessing a CRF and AP2 domain, but still lacked the TEH region and the MAPK phosphorylation site. The theoretical pI ranged from 4.56 to 9.93. As transcription factors, CRF proteins could rapidly accumulate in the nucleus in response to cytokinin [7]. The predicted subcellular localization of most of the *Br*CRFs was in the nucleus, except for the *Br*CRF9-12 (mitochondrial) and *Br*CRF21 (chloroplast). Gene structure revealed that the whole *BrCRFs* had almost no introns with just two exceptions (*BrCRF15* and *BrCRF21*) that contained a single intron. It was one of the representative characteristics of the ERF subfamily members [5], [24], [30].

**Table 2:** Characterization of *CRFs* in *B. rapa*.

Gene^a^	Locus^b^	Chr^c^	Subgenome^d^	Position^e^ (5′-3′ bp)	ORF length^f^ (bp)	Deduced polypeptide^g^			Subcellular Localization^h^
						Length (aa)	MW (kDa)	PI	
*BrCRF1*	Bra003462	A07	MF2	13317499–13318350(+)	852	283	31.8	5.43	n/0.88
*BrCRF2*	Bra004548	A05	LF	758917–759726(−)	810	269	30.0	7.62	n/0.97
*BrCRF3*	Bra022643	A02	MF1	8321229–8322287(+)	1059	352	39.7	4.56	n/0.30
*BrCRF4*	Bra003068	A10	LF	5480642–5481736(−)	1095	364	41.4	4.71	n/0/96
*BrCRF5*	Bra029079	A03	MF2	6246190–6247221(+)	1032	343	39.0	4.60	n/0.30
*BrCRF6*	Bra010389	A08	MF2	14241344–14242330(+)	987	328	37.4	4.81	n/0.60
*BrCRF7*	Bra040839	A08	MF2	12067512–12068489(−)	978	325	36.9	4.84	n/0.96
*BrCRF8*	Bra026295	A01	LF	10173842–10174855(−)	1014	337	38.2	4.96	n/0.98
*BrCRF9*	Bra007963	A02	MF1	12146525–12147028(+)	504	167	18.6	9.58	mms/0.49
*BrCRF10*	Bra003936	A07	MF2	15836513–15837010(−)	498	165	18.5	9.69	mms/0.63
*BrCRF11*	Bra016367	A08	MF2	18075909–18076385(+)	477	158	17.3	9.79	mms/0.63
*BrCRF12*	Bra012352	A07	MF1	8291523–8291966(+)	444	147	16.1	9.93	mms/0.63
*BrCRF13*	Bra013730	A01	LF	7472252–7473199(+)	948	315	34.9	5.34	n/0.70
*BrCRF14*	Bra019270	A03	MF1	25342408–25343340(+)	933	310	34.5	5.38	n/0.70
*BrCRF15*	Bra000736	A03	MF1	12751719–12752620(−)	822	273	29.9	5.12	n/0.70
*BrCRF16*	Bra004033	A07	MF2	16422562–16423515(+)	954	317	35.2	4.82	n/0.70
*BrCRF17*	Bra004318	A07	LF	17945569–17946483(−)	915	304	33.8	4.91	n/0.98
*BrCRF18*	Bra033923	A02	MF1	10922894–10923844(−)	951	316	34.7	4.62	n/0.70
*BrCRF19*	Bra025170	A06	LF	22970759–22971715(−)	957	318	35.2	5.03	n/0.98
*BrCRF20*	Bra036360	A02	MF1	22886535–22887380(−)	846	281	31.6	5.17	n/0.98
*BrCRF21*	Bra024743	A09	LF	5480642–5481736(−)	1005	334	37.4	5.17	cs/0.94

^a^ Names given to *BrCRFs* in this work.

^b^ Locus represented by the *B. rapa* genome database.

^c^ Chromosomal localization of the *BrCRFs*.

^d^ Three subgenomes in the *B. rapa* genome.

^e^ Position of *BrCRFs* on the chromosome, +/− represents the direction of transcription.

^f^ Length of open reading frame in base pairs.

^g^ Length (number of amino acids), molecular weight (kDa), and isoelectric point (pI) of the deduced polypeptide.

^h^ Localization predicted with PSORT. n, nucleus, mms, mitochondrial matrix space, cs, chloroplast stroma.

### Chromosomal distribution and duplications of *BrAP2/ERF* and *BrCRFs*

*Brassicaceae* genomes have undergone three rounds of whole genome duplication (WGD; hereafter referred to as 1R, 2R, and 3R, which are equivalent to the γ, β, and α duplication events, and *Brassica* genomes have undergone another whole genome triplication (WGT) after speciation from *Arabidopsis thaliana* at approximately 17–20 million years ago (MYA) [62]–[64], leading to significantly increased duplicated gene numbers in *B. rapa*. In plants, gene numbers are expanded by segmental and tandem duplication in gene families [65]. Gene duplication events are important to gene family evolution because duplicated genes provide the raw materials for the generation of new genes, which in turn facilitate the generation of new functions [66]. To further investigate the relationships between the genetic divergence within the AP2/ERF family and gene duplication in Chinese cabbage and Arabidopsis, the chromosomal location of each AP2/ERF gene was determined from the genomic sequences of *B. rapa* and *A. thaliana* (data not given). A total of 278 *BrAP2/ERF* genes were localized on the 10 chromosomes with an obviously uneven distribution. The three unmapped genes, *Bra040381*, *Bra040309*, and *Bra036016* were located on Scaffold000201, Scaffold000196, and Scaffold000111, respectively (Table S2). Tandem duplications and segmental duplications were identified (Table S3). Of the 281 *BrAP2/ERF* genes, we noted that 35 genes involved in tandem duplication events and 250 genes involved in segmental duplication events were observed, accounting for 12.5% and 89.0% of the total *BrAP2/ERF* genes, respectively. Comparatively, 30 genes involved in tandem duplication events and 75 genes involved in segmental duplication events were found among the 147 *AtAP2/ERF* genes, representing 20.4% and 51.0% of all the *AtAP2/ERF* genes (Table 3). The numerous duplicated genes supported that duplication events contributed largely to the current complexity of AP2/ERF genes both in the *A. thaliana* and *B. rapa*. It is noteworthy that the degree of segmental duplicated *AP2/ERF* genes in *B. rapa* is substantially higher than in *A. thaliana*, indicating whole genome triplication (WGT) of the *Brassica* genomes was mainly attributed to segmental duplication events. In Arabidopsis, about 75% of ERF genes, which lie within recently duplicated segmental chromosomes, have a clear relative in these regions and these duplicated pairs of ERF genes have been preferentially retained compared with other genes [62]. This finding was consistent with a previous report demonstrating that duplicated genes involved in signal transduction and transcription are preferentially retained [64], this could well explain the reason why a great quantity of AP2/ERF genes were retained during the long evolutionary history.

**Table 3:** Summary of the tandem duplicated and segmental duplicated genes of the AP2/ERF transcription factors in *Arabidopsis thaliana* and *Brassica rapa*.

Species	Total	Tandem duplicated genes		Segmental duplicated genes	
		Number	Percent (%)	Number	Percent (%)
*Arabidopsis thaliana*	147	30	20.4	75	51.0
*Brassica rapa*	281	35	12.5	250	89.0

Specifically, for *BrCRFs*, no tandem duplications were found, which was also observed among the known *CRFs* within sequenced genomes [31], whereas 7 pairs of segmental duplicates were present (Figure S2), indicating the expansion of *BrCRF* genes was also attributed to segmental duplication. Each pair of segmental duplicates was distributed on different chromosomes. Interestingly, *BrCRF7* did not form a pair of segmental duplicates with *BrCRF6* and *BrCRF8* even though it shares high similarity with them. Considering orthologs often retain equivalent functions during evolution [67], we examined the phylogenetic relationships between *BrCRFs* and *AtCRFs* using a local synteny-based method. The *Ka* and *Ks* values were determined (Table S4). Generally, each *BrCRF* has one to three putative orthologs in Arabidopsis. However, no *AtCRF9* orthologs were identified in Chinese cabbage. Additionally, *BrCRF7* had no orthologous Arabidopsis gene partner. These findings strongly indicate gene expansion and loss during the genome evolution among species.

### Evolutionary patterns and divergence of AP2/ERF genes in *B. rapa* and *A. thaliana*

**Figure 5:**
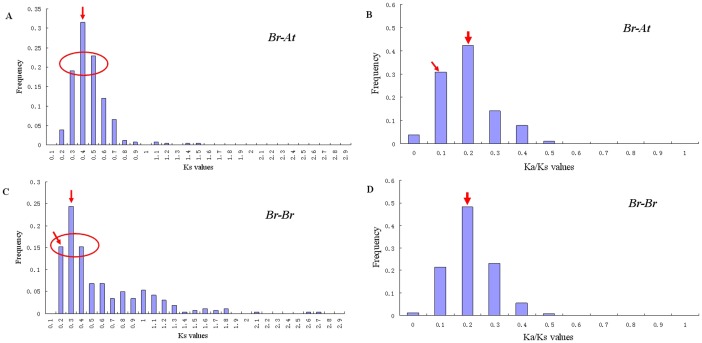
The *Ks* and *Ka/Ks* values distribution of the APE/ERF genes in the genome of *A.* *thaliana (At*) and *B. rapa* *(Br)* viewed through the frequency distribution of relative *Ka* and *Ks* modes. Distributions of *Ks* and *Ka/Ks* values were obtained from duplicated orthologous gene pairs (A, B) between the *Br* and *At* genomes and duplicated paralogous genes pairs (C, D) in the *Br* genome. The vertical axis indicates the frequency of paired sequences, whereas the horizontal axis denotes the *K*_s_ and *Ka/Ks* values with an interval of 0.1.

The *Ka* (non-synonymous substitution rates) and *Ks* (synonymous substitution rates) is a measure to explore the mechanism of gene divergence after duplication. Large-scale duplication events are defined as simultaneous duplications of genes. Assuming a molecular clock, the synonymous substitution rates (*Ks*) of these duplicates are expected to be similar over time. There are, however, substantial rate variations among genes [68]. We used the relative *Ks* measure as the proxy for time to evaluate the divergence time between *B. rapa* and *A. thaliana.* The frequency distributions of the relative *Ks* values obtained from duplicated orthologous gene pairs between the *B. rapa* and *A. thaliana* genomes and duplicated paralogous gene pairs in the *B. rapa* genome were shown in Figure 5 A, C. The relative *Ks* distribution of the duplicated orthologous gene pairs between the *BrAP2/ERF* and *AtAP2/ERF* genes peaked at 0.40–0.50, indicating that the AP2/ERF genes of the two genomes were separated approximately 27–33 MYA, which coincided well with previous studies of speciation between the *Brassica* and *Arabidopsis* genomes [69]–[72]. However, distinct peak for *B. rapa* genome was found at *Ks* = 0.30–0.40, suggesting a large-scale event 20–26 MYA for the *B. rapa* genome. This relatively smaller *Ks* value is responsible for the *Ks* between the paralogous gene pairs generated by WGT in *B. rapa* after the divergence from *Arabidopsis thaliana.* The ratio *Ka*/*Ks* provides a measure of the selection pressure to which a gene pair is subject. *Ka*/*Ks* = 1 means neutral selection, *Ka*/*Ks*<1 means purifying selection, and *Ka*/*Ks*>1 means accelerated evolution with positive selection [73]. We also obtained the *Ka*/*Ks* ratio from duplicated orthologous gene pairs (Figure 5 B) between the *B. rapa* and *A. thaliana* genomes and duplicated paralogous genes pairs (Figure 5 D) in the *B. rapa* genome. Both the *Ka*/*Ks* values for *Br-At* and *Br-Br* displayed a peak at 0.2–0.3, suggesting similar purifying selection for the two genomes. However, the frequency of *Ka*/*Ks* values at 0.1–0.2 was 30.7% for *Br-At* compared to 21.5% for *Br-Br*, indicating greater selective constraint for *Br-At*.

### Phylogenetic relationships of the *BrCRF* gene family

To classify subgroups and to uncover the evolutionary relationships between *BrCRFs* and *AtCRFs*, multiple alignment analyses were performed using both the complete amino acid sequences and the conserved amino acid sequences of the CRF and AP2 domains. Similar phylogenetic trees were generated (Figure 2, Figure S3). The 21 *BrCRFs* were divided into five clades: Clade I *(BrCRF13–BrCRF15*), Clade II (*BrCRF3–BrCRF8*), Clade III (*BrCRF1 and BrCRF2*), Clade IV (*BrCRF9–BrCRF12*) and Clade V (*BrCRF16–BrCRF21*). Based on the classification on protein level discussed previously, Clade I, Clade II, and Clade III were grouped to Type A, Clade IV was grouped to Type B, and Clade V belonged to Type C.

In addition, further phylogenetic reconstruction using complete CRF proteins sequences from several species containing *A. thaliana*, *B. rapa*, *S. lycopersicum*, *P. trichocarpa* (dicotyledons), *O. sativa, Z. mays* (monocotyledons) and *P. patens* (moss) was performed to confirm the *BrCRF* subgroups and to investigate the evolution of *CRFs* among species. According to the genetic relationships and sequences features, the tree was divided into three groups: Group A, Group B, and Group C. Group A could be further divided into CladeI, IIand III, Group B could be further divided into Clade IV, VI, VII, VIII and IX. Group C contained a single Clade V (Figure S4). The TEH region on the N-terminal ends and SP[T/S]SVL motif on the C-terminal ends are typical features for some CRFs. Previous research showed that the classification of CRFs into Clades A and B coincided with the presence or absence of the TEH region [31]. Zwack et al. delineated CRFs into five distinct Clades (I–V), each of which was best defined by a unique and highly conserved C-terminal sequence [57]. In our research, the correlation between both the TEH region and SP[T/S]SVL motif with divergence of the CRFs was investigated. Both the TEH region and the SP[T/S]SVL motif were indispensable for the classification of CRFs (Table S5). Group A contained only the dicotyledons and CRFs in this group all contained the TEH sequence and the SP[T/S]SVL motif. CRFs in Clades V with rather distant phylogenetic relationships from other Groups were totally absent of the TEH sequence and the SP[T/S]SVL motif. Group B was mainly composed of the monocotyledons and moss, and lacked the TEH sequence and the SP[T/S]SVL motif except for partial members in Clade VI. The SP[T/S]SVL motif seemed more prevalent than the TEH sequence because a few CRFs had the SP[T/S]SVL motif but lacked the TEH sequence, especially the CRFs in rice and maize. Furthermore, more CRFs in dicotyledons had the TEH sequence than that in monocotyledons, with more than half of the CRFs in dicotyledons containing the TEH sequence, whereas only one CRF in rice and maize were found with the TEH sequence. Both the TEH sequence and the SP[T/S]SVL motif were absent in lower plants such as *P. patens*. The evolution and functions of TEH sequence and SP[T/S]SVL motif are still waiting to be clarified.

### Analysis of the putative promoter regions of *BrCRFs*

*Cis*-regulatory elements, which are located upstream of genes and act as binding sites for TFs, have essential roles in determining the tissue-specific or stress-responsive expression patterns of genes [74]. Increasing evidence shows that multi-stimulus responsive genes are closely correlated with *cis*-regulatory elements in the promoter regions [75], [76]. To further understand transcriptional regulation and the potential functions of these genes, 2,000 bp putative promoter regions upstream of the transcriptional start site were applied to identify putative stress-responsive *cis*-regulatory elements [77]. A number of abiotic stress elements were found (Table S6). Three drought-stress *cis*-elements (S000176, S000408, and S000415), one salt-stress (S000453), one heat-stress (S00030), one cold-stress (S000407), and one wound-stress (S000457) widely occur in the promoter regions of *BrCRFs*, which presented clues that *BrCRFs* might be closely related with abiotic stress and have potential functions in the abiotic stress tolerance. In particular, *BrCRF7* and *BrCRF13* possessed up to 20 and 22 drought-stress elements (S000415), whereas *BrCRF13* and *BrCRF14* had up to 22 and 20 cold-stress elements (S000407), respectively.

In addition, to investigate possible biological functions and regulatory mechanisms of *BrCRFs*, the 2,000 bp putative promoter regions upstream of the transcriptional start site were examined for conserved motifs using MEME analysis [44] (Figure S5). Two highly conserved TC-rich regions (Motif 1, 3) and AG-rich regions (Motif 4, 5) were significantly enriched in the promoters of all 21 *BrCRFs*. Motif 2 and Motif 6 (AC-rich region) was also found across 20 *BrCRFs* and 18 *BrCRFs*, respectively. The gene ontology of the motifs was determined using GOMO analysis to interpret the motifs. According to the prediction (Table S7), *CRFs* were involved as transcription factors in various events in plants, including biological processes, cellular component, and molecular functions during vegetative and reproductive development. Previous studies showed that *CRF* expression could be regulated by various hormones, such as cytokinin, ethylene, methyl jasmonate, and salicylic acid [78]. MEME analysis consistently revealed that the *BrCRFs* could be involved in cytokinin- and abscisic acid–mediated signaling pathways. *BrCRFs* also responded to jasmonic acid, cytokinin, salicylic acid, and auxin stimuli.

### Expression profiles of *BrCRFs* in various tissues and organs

**Figure 6:**
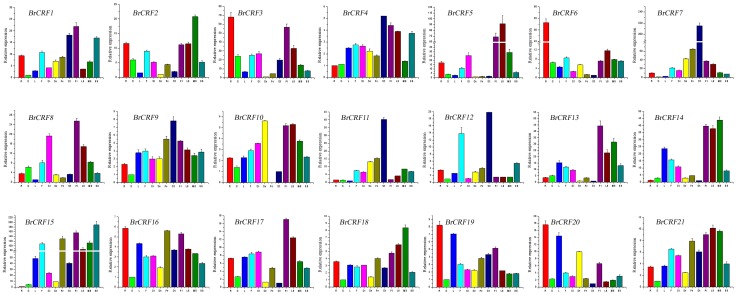
Relative expression profiles of *BrCRFs* in various tissues and organs. R, roots; S, floral stems; L, leaves; F, flowers; Si, immature siliques; Se, sepals; Pe, petals; St, stamens; Pi, pistils; LB, little buds; MB, middle buds; and BB, big buds. Bars showed ±SE of the mean of two biological and three technical replicates.

Tissue-specific and developmental stage–related expression data are useful in identifying genes involved in defining the precise nature of individual tissues in a given developmental stage [74]. To obtain a first glance of the roles of each of the *BrCRF* during the vegetative and reproductive development, qRT-PCR was used to analyze the transcription levels of these genes in the roots, stems, leaves, flowers, immature siliques, sepals, petals, stamens, pistils, little buds, middle buds, and big buds. Generally, the 21 *BrCRFs* were expressed in different plant tissues and organs to varying degrees (Figure 6). The *BrCRF* expression levels were consistent across the plant tissues and organs. However, some genes showed preferential tissue and organ expression. Comparatively, *BrCRF6*, *BrCRF19*, and *BrCRF20* had relatively higher expressions in vegetative organs, whereas *BrCRF7*, *BrCRF8*, *BrCRF11*, and *BrCRF12* showed major transcripts in reproductive organs. *BrCRF3* and *BrCRF6* had specifically higher expressions in the roots; *BrCRF19* and *BrCRF20* were highly expressed in both the roots and the leaves. In four whirl flower organs, *BrCRF4*, *BrCRF*7, *BrCRF11*, and *BrCRF12* were expressed at very high levels in stamens, whereas *BrCRF3*, *BrCRF5*, *BrCRF8*, *BrCRF13*, *BrCRF14*, *BrCRF15*, and *BrCRF17* were expressed abundantly in the pistils. In the floral buds, *BrCRF5*, *BrCRF8*, *BrCRF13*, *BrCRF14*, and *BrCRF15* showed relatively higher expressions. The *BrCRF8* transcripts were highest in the siliques. Though segmental duplicated gene pairs originate from common ancestor genes, their expression profiles are not always the same. Among the seven segmental duplicated gene pairs, only three pairs (*BrCRF11* and *BrCRF12*, *BrCRF13* and *BrCRF14*, and *BrCRF19* and *BrCRF20*) showed similar expression patterns in various tissues and organs. After the whole genome triplication (WGT) of *B. rapa* with gene expansion, these expanded genes probably evolved into independent new genes with irreplaceable functions, leading to changes in expression patterns.

### Expression profiles of *BrCRFs* under drought and salt stress

**Figure 7:**
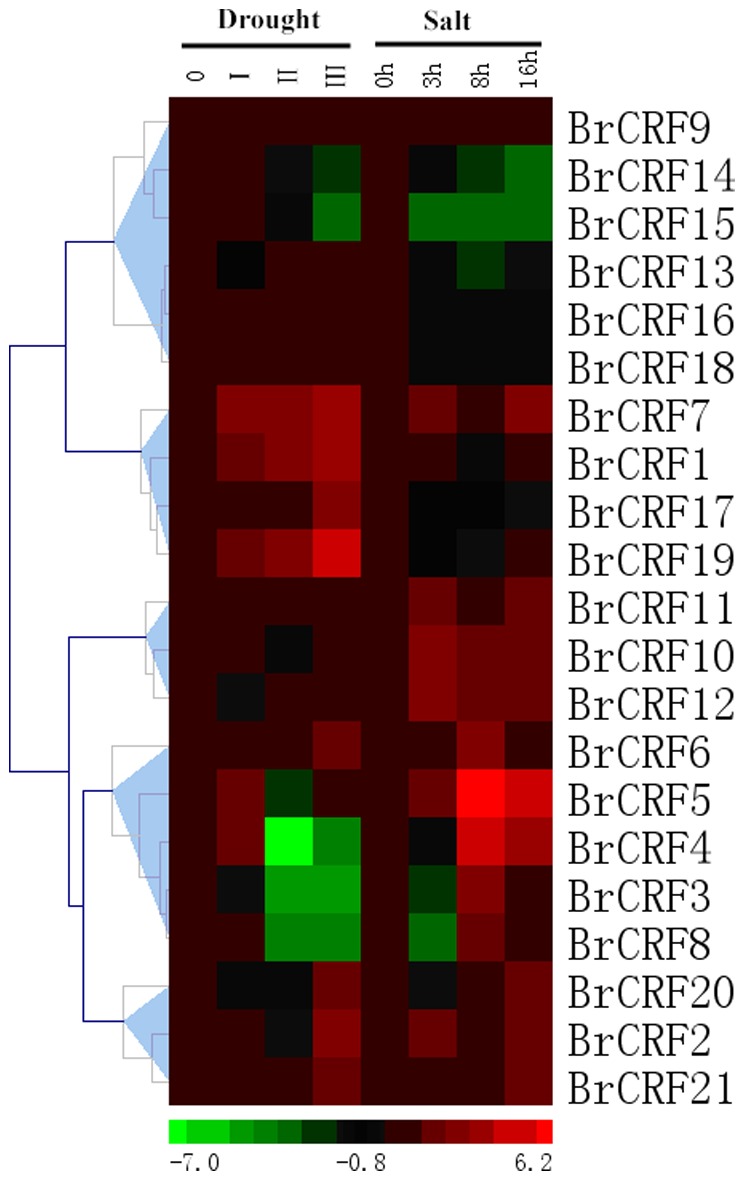
The heat map shows the real-time quantitative RT-PCR (q-RT-PCR) analysis results of *BrCRF* genes under drought and salt treatments with two biological and three technical replicates. The expression levels of genes are presented using fold-change values transformed to Log_2_ format compared to control. The Log_2_ (fold-change values) and the color scale are shown at the bottom of heat map.

To date, many AP2/ERF TFs from various plant species have been shown to be involved in abiotic stress responses [79]. As an important part of the AP2/ERF family, ERF TFs include members that respond to various abiotic stresses, such as drought and high salinity [80], [81], and confer stress tolerance through overexpression in transgenic plants [81]. As *CRFs* locate in two subgroups (Group VI and VI-L) of the ERF subfamily, *CRFs* also share the properties to respond to abiotic stresses. In fact, expression analysis during salt treatment (200 mM NaCl) revealed induction of *SlCRF1*, *SlCRF4*, and *SlCRF6* at both 1 h and 3 h, as well as a minor induction of S*lCRF2*, *SlCRF5*, and *SlCRF7* at 3 h [78]. Theoretically, *BrCRFs* also respond to various abiotic stresses because of the presence of stress-inducible *cis*-regulatory elements in the promoter regions. qRT-PCR was used to analyse the expression profiles of *BrCRFs* under drought and salt stress conditions. The data were presented with clusters using fold-change values transformed to Log_2_ format. As shown in Figure 7, most of the *BrCRF* transcripts were upregulated under drought and saline conditions and the genes in same Clade shared similar response patterns. For the drought treatment, *BrCRF1* and *BrCRF2* in Clade III were continuously induced and kept a high level in seriously drought conditions. For genes in Clade II, *BrCRF3*, *BrCRF4*, *BrCRF5*, and *BrCRF8* were primarily induced and upregulated, but the number of transcripts decreased under more severe drought conditions, even decreasing to undetectable levels. However, the expression of *BrCRF6* and *BrCRF7* was upregulated to varying degrees. Drought treatment resulted in minor effect on genes in Clade IV, only *BrCRF12* showed slightly elevated expression levels. In Clade I, *BrCRF15* and *BrCRF16* were primarily induced and upregulated, but their transcripts dropped below the basal level, whereas *BrCRF14* showed opposite response. The six members in Clade V were induced and upregulated at different degrees, *BrCRF19* showed especially 30-fold increase in expression levels. As for the salt treatment, similar response mechanism occurred. Transcripts of *BrCRF1* and *BrCRF2* in Clade III initially increased and then dropped, but increased again at 16 h with relatively small amplitudes of variation. For genes in Clade II, the expression of *BrCRF3*, *BrCRF4*, and *BrCRF8* were downregulated after 3 h and upregulated after 8 h, and then returned to basal levels or relatively high levels after 16 h. *BrCRF5* showed more than a 70-fold increase in expression levels after 8 h and maintained an approximately 20-fold increase in transcripts after 16 h. *BrCRF10* and *BrCRF12* in Clade IV were primarily induced and upregulated, and their expression levels dropped, but stayed at a relatively high levels after 8 h and 16 h. The expression levels of *BrCRF9* and *BrCRF11*, did not change significantly under salt treatment. High salinity suppressed the expression of *BrCRF13*, *BrCRF14*, and *BrCRF15* in Clade I. The transcripts of *BrCRF16*, *BrCRF17*, and *BrCRF18* primarily dropped, but returned to basal levels after 16 h. Both *BrCRF19* and *BrCRF20* showed decreased expression levels, which then increased, and maintained relatively high levels. *BrCRF21* were continuously induced with treatment time going on. Although the functions of *BrCRFs* in abiotic stress responses are still largely unknown, they are likely involved in gene regulation under stress conditions. They are likely to regulate the developmental, physiologic, and biochemical responses of plants to a variety of environmental stress conditions, thereby increasing the stress tolerance of Chinese cabbage.

### Effects of exogenous 6-BA, NAA, and ABA on the expression of *BrCRFs*

**Figure 8:**
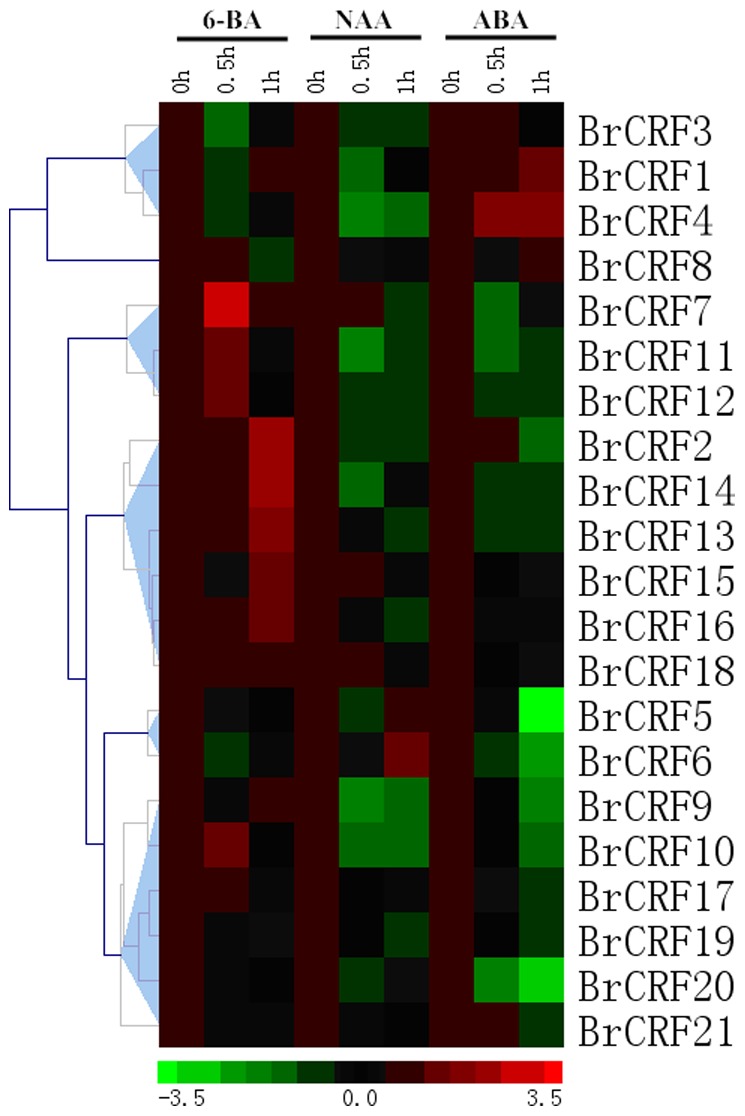
The heat map shows the real-time quantitative RT-PCR (q-RT-PCR) analysis results of *BrCRF* genes with exogenous 6-BA, NAA, and ABA treatments with two biological and three technical replicates. The expression levels of genes are presented using fold-change values transformed to Log_2_ format compared to control. The Log_2_ (fold-change values) and the color scale are shown at the bottom of heat map.

Previous research showed that *AtCRFs* and *SlCRFs* are implicated in responses to various plant hormones, especially cytokinin. Analysis of the promoter regions of *BrCRFs* using the MEME suite indicated that *BrCRFs* are involved in cytokinin and abscisic acid–mediated signaling pathway, and in response to jasmonic acid, cytokinin, salicylic acid, and auxin stimuli. The response patterns of *BrCRFs* to plant hormones were examined using qRT-PCR analyses. The data were also presented with clusters using fold-change values transformed to Log_2_ format. Viewing from Figure 8, most of the *BrCRFs* responded to 6-BA, NAA, and ABA to varying degrees. For 6-BA treatment, the results generally followed a pattern similar to the proposed cytokinin induction mode with each specific Clade of *CRFs* in Arabidopsis and tomato [57] (Figure 8). *BrCRF2* in Clade III and *BrCRF13*, *BrCRF14*, and *BrCRF15* in Clade I showed continuous elevated expression levels, which was consistent with the cytokinin expression characteristics for *CRFs* in Clades I and III. However, *BrCRF7* was strongly induced initially by cytokinin but its transcript returned to the basal level after 1 h. *BrCRF10*, *BrCRF11*, and *BrCRF12* shared similar expression patterns with *BrCRF7*, they just showed relatively weaker induction levels after 30 min. The other remaining genes showed minor or no alterations with CK treatment. More than 20 auxin-related genes could be regulated by cytokinin, indicating that auxin–cytokinin crosstalk might be realized through transcriptional regulation [33]. Under treatment with auxin, a CK antagonist, the majority of *BrCRF* genes were subjected to negative regulation within our expectations. Only one gene, *BrCRF6*, showed significantly upregulated expression (about 2.7-fold) after 1 h. ABA is a phytohormone that regulates a variety of growth and developmental processes, including seed development, dormancy, germination, and stomatal movement [80], [82]. Furthermore, ABA is extensively involved in responses to abiotic stresses such as drought and high salinity and osmotic stress [82], [83]. As important AP2/ERF TFs, *CRFs* were broadly expressed in various tissues and organs, and closely linked with abiotic stresses. Thus, *CRFs* may be also involved in ABA homeostasis. Consistently, *BrCRF1*, *BrCRF4*, and *BrCRF8* were induced by ABA treatment, whereas *BrCRF5*–*BrCRF7*, *BrCRF9–BrCRF14*, and *BrCRF20* exhibited prominently suppressed transcript levels. *BrCRF2* and *BrCRF21* were primarily induced and upregulated, but then their transcriptions dropped below the basal level. The homeostasis mechanism among various plant hormones is complex, and a deeper understanding of cross-talk between *CRFs* and hormones is necessary.

## Discussion

The AP2/ERF family is a large group of TFs involved in plant development and plant abiotic stress responses (Table S8). To date, AP2/ERF genes have been identified and characterized in various plant species. A total of 281 AP2/ERF family members have been identified in Chinese cabbage, including 131 ERF genes, 105 DREB genes, 30 AP2 genes, 14 RAV genes, and 1 soloist. AP2/ERF TFs in Chinese cabbage are about 1.9-fold more than those in *A. thaliana* (147), with a higher proportion than non-TFs [62]. *Brassica* genomes have undergone whole genome triplication (WGT) after speciation from *Arabidopsis thaliana*, leading to significantly increased duplicated genes. Up to 35 tandem duplicated genes and 252 segmental duplicated genes were found among the 281 *BrAP2/ERF* TFs, while 30 tandem duplicated genes and only 75 segmental duplicated genes were found in *A. thaliana*, suggesting that the expansion of *BrAP2/ERF* TFs after speciation from *Arabidopsis thaliana* is mainly attributed to segmental duplication events during the whole genome triplication (WGT). Duplication occurs in an individual, and can be fixed or lost in the population, similar to a point mutations [84]. Unless the presence of an extra amount of gene product is advantageous, two genes with identical functions are unlikely maintained in the genome [85]. However, several authors have provided evidence that genes involved in transcriptional regulation, response to biotic stimuli, and signal transduction have been preferentially retained following genome duplications [86]–[88]. Duplication events within a genome usually produce paralogs, and these genes may perform part of the original function (subfunctionalization) or even new functions (neofunctionalization) if they are not silenced (non-functionalization) [89]. We examined the relative expression profiles of *BrCRFs* in various tissues and organs, as well as the expression patterns of *BrCRFs* in response to salt, drought, and exogenous plant hormones. The results show that even though segmental duplicated gene pairs originate from common ancestor genes, their expression profiles are not always the same. These expanded genes have probably evolved into independent new genes with irreplaceable functions; thus, they were preserved.

To determine the relative divergence of the respective lineages, we analysed the *Ka* and *Ks* modes of the duplicated paralogous and orthologous gene pairs. Using the commonly adopted estimate of mutational rate of 1.5×10^−8^ synonymous substitutions per site per year [54], we estimated the times of lineage divergence. Based on the frequency distributions of the relative *Ks* values for *Br*-*At* and *Br*-*Br*, we estimated that *B. rapa* diverged from *A. thaliana* at approximately 27–33 million years ago after the third whole genome duplication (3R) event, and another large-scale event occurred around 20–26 million years ago for the *B. rapa* genome, just coincident with the time of the third polyploidy event (4R), a *Brassica* lineage-specific whole genome triplication after the split of *Brassica* from the common ancestor of *Brassica* and *Arabidopsis* [90]. The *Ka*/*Ks* ratio provides a sensitive measure of selective pressure on the protein. Most amino acids in a functional protein are under affects only a few sites at a few time points. Therefore, positive selection was thought to be one of the major forces in the emergency of new motifs/functions in protein after gene duplication [91]. The gene pair is said to be under ‘purifying selection’ if *Ka*/*Ks*<1: some replacement substitutions have been purged by natural selection, presumably because of their deleterious effects. The smaller the *Ka*/*Ks* ratio is, the greater the number of eliminated substitutions and the greater the selective constraint under which the two genes have evolved [92]. Both the *Ka*/*Ks* ratio for *Br-At* and *Br-Br* displayed a peak at 0.2–0.3, suggesting purifying selection for the AP2/ERF genes and they had undergone substitutions elimination and great selective constraint during the long evolutionary history by natural selection.

Cellular localization is often an important factor in determining protein function. In most cases, TFs are located only in the nucleus after they have been synthesized in the cytoplasm. However, in some cases, TFs are located in different compartments of the cell. The ability of proteins to localize into more than one cell compartment is called dual targeting and can be regarded as post-translational regulatory mechanism [93]. PSORT was applied to predict and determine the localization of 21 *Br*CRFs. Most *Br*CRFs showed nuclear localization with several exceptions located in the mitochondria or chloroplast. CRF proteins were uniformly located in the cytoplasm of Arabidopsis protoplasts and they could rapidly accumulate in the nucleus in response to cytokinin [7]. However, contradictorily, Ketelsen revealed that *At*CRF5 has a strong nuclear localization and weak cytoplasmic localization; thus, *At*CRF5 is not dually targeted. Ketelsen fused GFP to the C-terminal end of *At*CRF5 (CRF5::GFP) whereas Rashotte constructed a fusion protein with the GFP tag located at the N-terminus (GFP::CRF5) (Bernd Ketelsen, unpublished PhD thesis). Organelle import is mostly facilitated by presequences or transit peptides located at the N-terminus of proteins that function as import addresses. Fusion with GFP at this end essentially inhibits its function as an import signal [94]. Fusing GFP with the C-terminus might prevent CRF5 to localize naturally or vice versa (Bernd Ketelsen, unpublished PhD thesis). The subcellular localization of *Br*CRFs is still to be determined.

AP2/ERF proteins are plant-specific TFs that have been found in the green alga *Chlamydomonas reinhardtii* [95]. However, Magnani et al. found homologs in a cyanobacterium, in a ciliate, and in two viruses. In these organisms, the proteins are predicted to be HNH-endonucleases. AP2/ERF proteins in plants have been hypothesized to originate from these organisms and were introduced into plants via lateral gene transfer [96]. Furthermore, to understand how they evolved in plant history, Mizoi et al. analyzed the phylogenetic relationships of AP2/ERF TFs that belong to the four major subfamilies, including those from *Arabidopsis thaliana*, *Selaginella moellendorffii*, *Physcomitrella patens*, and *Chlamydomonas reinhardtii*, which represented angiosperms, lycophytes, mosses, and green algae, respectively. Detailed investigation revealed that they were established in a common ancestor of extant mosses and vascular plants [79]. More significance has been recently attached to the newly born CRF protein members, a subset of the AP2/ERF superfamily. CRF proteins have already been identified in liverworts, mosses, lycopods, ferns, conifers, and all major lineages of flowering plants. However, no CRF domain–containing genes were found in any species of green algae including the completely sequenced genomes of *Chlamydomonas*, *Micromonas* (2 species) and *Ostreococcus*, despite the presence of clearly identifiable AP2/ERF domain proteins [31]. This finding suggests that the occurrence of AP2 DNA binding domain predates the CRF domain. The functions of specific CRF domain remain mostly elusive. The CRF domain may function as a protein–protein interaction domain, allowing CRF domain–containing proteins to form heterodimers or homodimers with each other or with themselves [97]. Additionally, Arabidopsis CRFs (CRF1–CRF8) are able to interact directly with almost all Arabidopsis AHPs (AHP1–AHP5), thereby indicating CRFs may act as a potential branch of the cytokinin signaling pathway [97]. The CRF domain may also be connected to biotic or abiotic stress resistance. *AtCRF5* overexpression confers pathogen resistance to Arabidopsis plants [98]. *Pti6* overexpression in tomato confers increased pathogen resistance [81] and *Tsi1* overexpression in tobacco enhances resistance to pathogen attack and osmotic stress [81]. However, a large proportion of non-CRF AP2/ERF genes also participate in responses to various biotic and abiotic stresses [17], [18], [99]–[101], suggesting the involvement of the AP2 domain in these processes. Therefore, a more detailed analysis is necessary to demonstrate the unique roles of the CRF domain.

Extensive research has confirmed that AP2/ERF TFs are involved in plant growth and development, hormone response, and biotic or abiotic stress responses. Xu et al. collected 70 ERF genes identified in various plants and classified them into 8 clusters (I to VIII) and 14 subclusters based on phylogenetic relationships, gene structures, conserved motifs, and biologic functions, indicating that ERF genes, especially those in Cluster II and VII could improve plant resistance because their overexpression enhanced resistance to various diseases and improved tolerance to drought, salt, and freezing in transgenic plants [102]. Mizoi et al. discussed the functions of the each AP2/ERF-type TF in plant abiotic stress responses, with special emphasis on the regulation and function of two major types of DREBs, DREB1/CBF and DREB2 [79]. Moreover, it is important to note that increasing evidence proves that CRFs, a subset of AP2/ERF proteins, also regulate a variety of developmental processes and stress responses in plants. In Arabidopsis, *CRF2* participates in the signal transduction of cytokinin to induce chloroplast division [9]. Many *CRFs* in Arabidopsis and tomato are engaged in cytokinin and ethylene response, and salt treatment (NaCl) induces about half of the *SlCRFs* to some degree [78]. In the present research, 21 *BrCRFs* were broadly expressed in various plant tissues and organs, with some genes showed preferential expression in specific tissues and organs. In addition, these genes responded well to salt and drought conditions, and treatment with exogenous 6-BA, NAA, and ABA. Therefore, determining the potential values of *CRFs* may bring new surprises for us.

Chinese cabbage (*Brassica rapa* ssp. *pekinensis*) is an important vegetable widely cultivated in Asia, especially in China, Korea, and Japan. Like many other crops, Chinese cabbage is challenged by abiotic stress, such as drought, high salinity, and adverse temperature [25]. AP2/ERF proteins and CRF TFs are excellent candidates for improving crop resistance. We have established a highly efficient transformation system with leaf disk in Chinese cabbage, we are optimistic to reveal the functions of *BrCRFs* and obtain stress-resistant crops *via* genetic improvement.

## Supporting Information

Figure S1 **Alignment of the conserved amino acid sequences of each AP2/ERF subfamily group from** ***B. rapa*** **.** (A) Alignment of the AP2/ERF domains and additional domains of each DREB subfamily group (GroupI∼IV) from *B. rapa*. (B) Alignment of the AP2/ERF and additional domains of each ERF subfamily group (GroupV∼XI) from *B. rapa*. (C) Alignment of the AP2/ERF domains of AP2 subfamily from *B. rapa*. (D) Alignment of the AP2/ERF and B3 domains of RAV subfamily from *B. rapa*. The black background represented the most conserved amino acid residues in each group. The black bar and arrows represented predicted α-helix and β-sheet regions, respectively, within the AP2/ERF domain. Asterisks represented amino acid residues that directly make contact with DNA.
(DOC)

Figure S2 **Synteny analysis of** ***BrCRFs*** **in ±100kb region with score greater than 1500.** Synteny analysis revealed evidence of the segmental duplication among *BrCRFs*.
(DOC)

Figure S3 **Neighbor joining tree of CRF proteins based on conserved CRF and AP2 domains of** ***Br*** **CRFs with their Arabidopsis counterparts.** The tree could be divided into 5 Clades, Clade I, II, III, IV and V.
(DOC)

Figure S4 **Neighbor joining tree of CRF proteins from 81 sequences identified by genus name and numerical identifiers from respective databases.** The phylogenetic tree of CRF gene family contained *A. thaliana* (12), *B. rapa* (21), *O. sativa* (9), *Z. mays* (9), *S. lycopersicum* (12), *P. trichocarpa* (11) and *P. patens* (7). The tree could be divided into 3 groups, Group A, Group B and Group C. Besides, Group A could be further divided into Clade I, II and III, Group B could be further divided into Clade IV, VI, VII, VIII and IX. Group C contained a single Clade V.
(DOC)

Figure S5 **Motifs found with putative promoter regions of** ***BrCRFs*** **by MEME analysis.**
(DOC)

Table S1 **Forward and reverse primers used in the qRT-PCR gene expression studies.**
(DOC)

Table S2 **Summary of the AP2/ERF family transcription factors identified in** ***B. rapa*** **.**
(XLS)

Table S3 **Summary of the duplicated gene pairs and determination of the** ***Ka*** **,** ***Ks*** **values of the AP2/ERF family transcription factors in** ***B. rapa*** **and** ***A. thaliana*** **.**
(XLS)

Table S4 ***Ka*** **and** ***Ks*** **values of** ***CRF*** **orthologs between** ***B. rapa*** **and** ***A. thaliana*** **.** The *BrCRFs* and *AtCRFs* were both divided into 5 Clades based on the phylogenetic trees previously described. Values of *Ka* and *Ks* were determined. No orthologs of *AtCRF9* were identified in *B. rapa.* Likewise, no orthologs of *BrCRF7* existed in *A. thaliana.*
(DOC)

Table S5 **Analysis of the TEH sequence in N-terminal ends and Put MAPK Phos (Putative MAPK phosphorylation site) in C-terminal ends of several plant CRF family proteins.** Proteins lacking both the TEH sequence and Put MAPK Phos were not listed. — represented no corresponding sequences existed.
(DOC)

Table S6 **Summmary of abiotic-stress inducible** ***cis*** **-elements in the putative promoters of** ***BrCRFs*** **.** *Cis*-elements with larger numbers were marked red.
(DOC)

Table S7 **Gene ontology for motifs by GOMO analysis.** BP stands for biological process, CC stands for cellular component and MF stands for molecular function.
(DOC)

Table S8 **Summary of AP2/ERF family genes whose biological functions have been reported.**
(DOC)
