# Supplementary material for: Genome-Wide Identification, Phylogeny, Evolution and Expression Patterns of AP2/ERF Genes and Cytokinin Response Factors in Brassica rapa ssp. pekinensis
Source: PLoS One. 2013 Dec 30;8(12):e83444. doi: 10.1371/journal.pone.0083444 (PMC3875448; doi:10.1371/journal.pone.0083444)

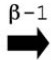

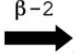

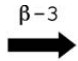

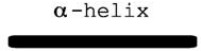


**Ⅰ**


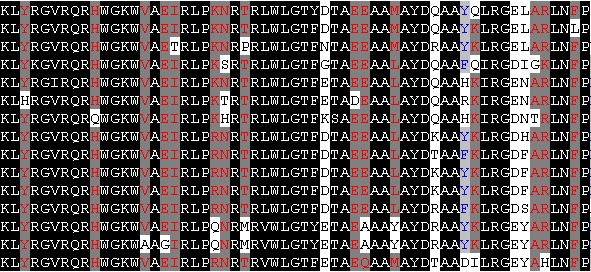

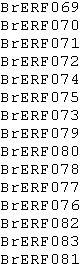


**AP2 domain**

★

★

★

★

★

★

★

**LxxxvVxxKLxxI**

**LxKxPSxxIDxxWxxI**


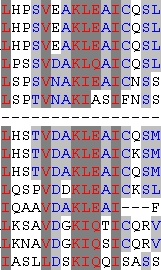

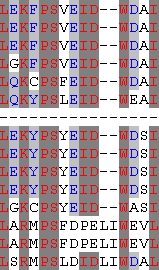


**Ⅱ**


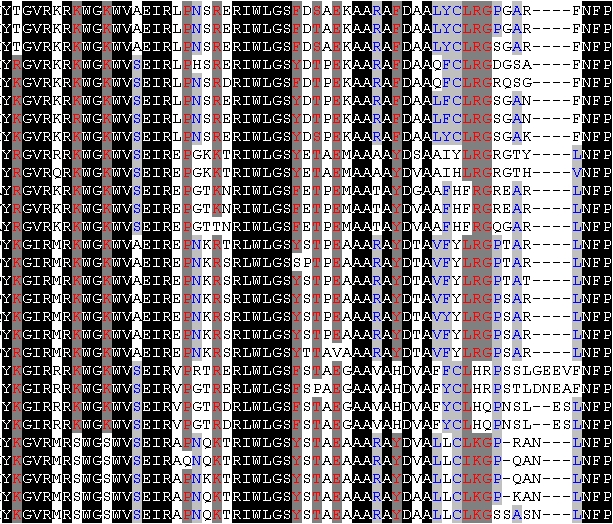

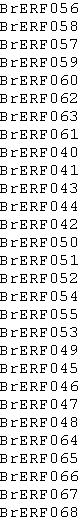


**AP2 domain**


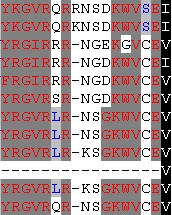

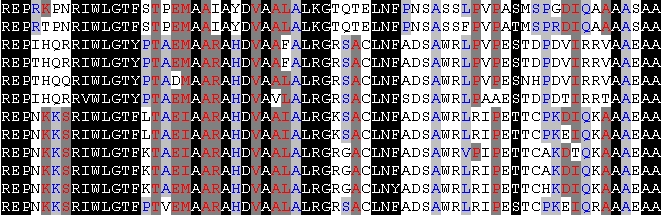

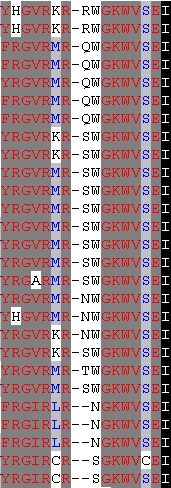


**Ⅲ**


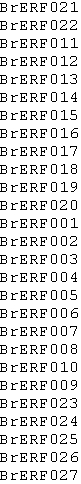

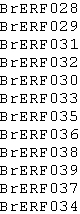

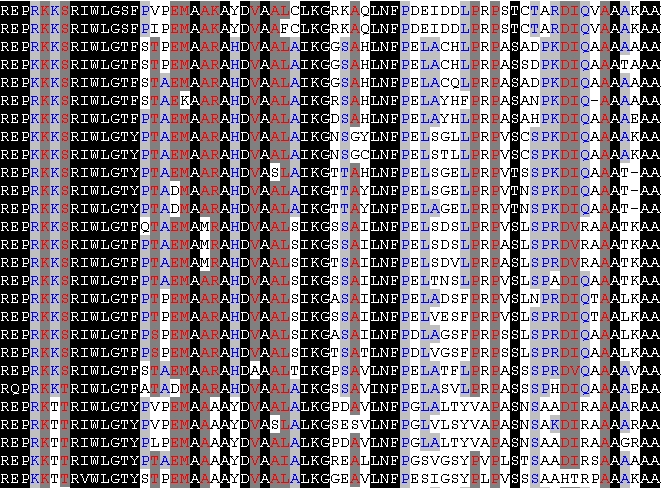


**AP2 domain**

**A rich**

**LPxL**


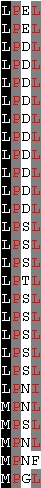


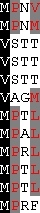


**KKGCMKGKGGPEN**

**AP2 domain**


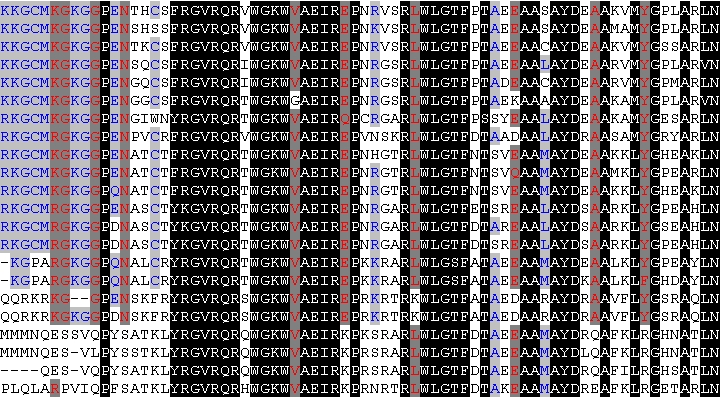

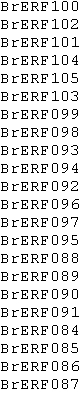


**Ⅳ**

**Figure S1A**

Alignment of the AP2/ERF domains and additional domains of each DREB subfamily group (GroupⅠ~Ⅳ) from *B. rapa.* All groups had representative AP2 domains. Additionally, Group Ⅰhad another two conserved consensus sequences, Group Ⅲ had an A-rich region and consensus sequences [LPxL], Group Ⅳ also had one conserved consensus sequences. The black background represented the most conserved amino acid residues in each group. The black bar and arrows represented predicted α-helix and β-sheet regions, respectively, within the AP2/ERF domain. Asterisks represented amino acid residues that directly make contact with DNA (Allen et al., 1998).


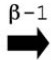

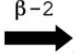

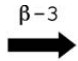

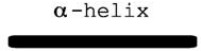


**AP2 domain**

★

★

★

★

★

★

★

**QMIEELL**


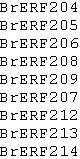

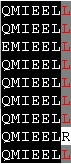

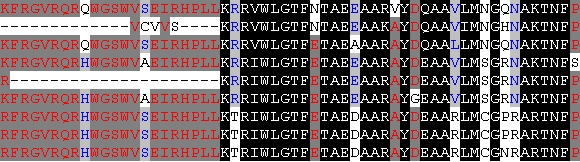


**Ⅴ**

**CRF domain**


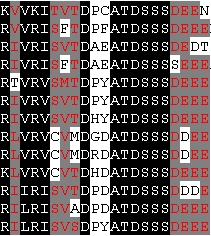


**AP2 domain**


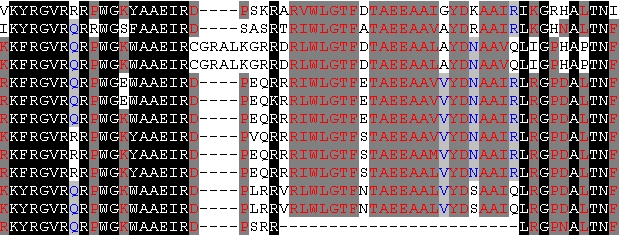


**MAPK**

**TEH**


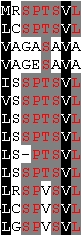

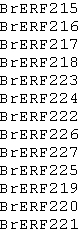

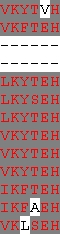


**Ⅵ**

**CRF domain**


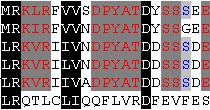


**AP2 domain**


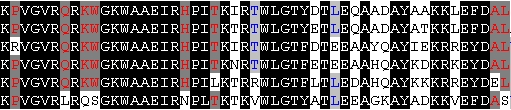


**LPDxDFxD**

**FNxxxLxIP**


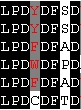

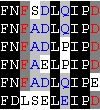


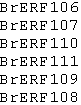


**Ⅵ-L**

**AP2 domain**


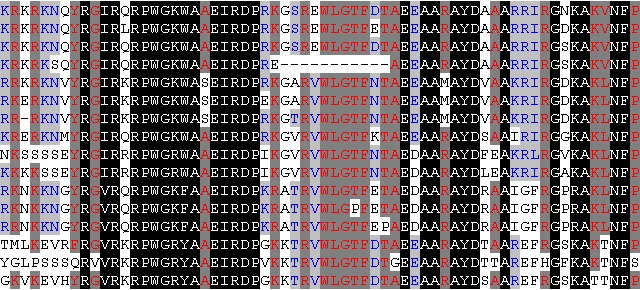

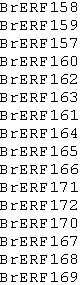


**Ⅶ**


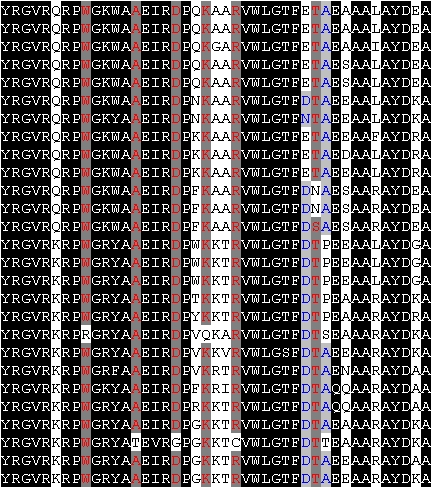

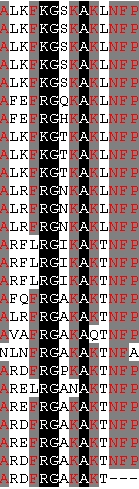


**Ⅷ**


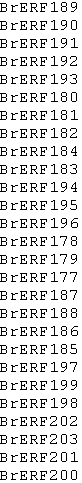

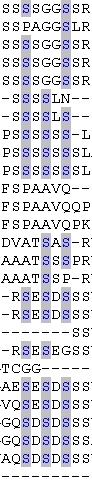


**AP2 domain**

**Ser rich region**

**AP2 domain**


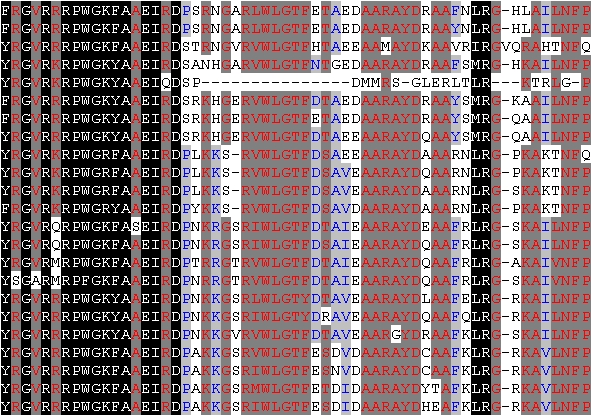


**Ⅸ**


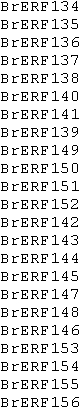


**AP2 domain**


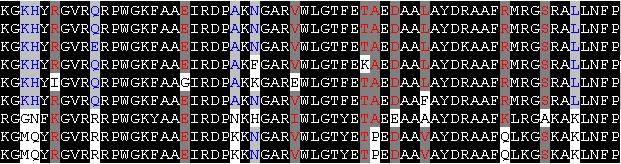


**Ser rich region**

**Ser rich region**


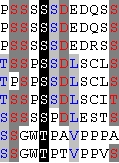

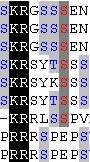


**Ⅹ**


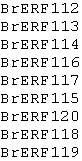

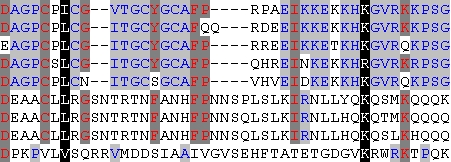

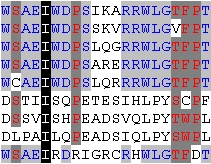


**AP2 domain**


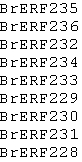


**Ⅹb-L**

**AP2 domain**


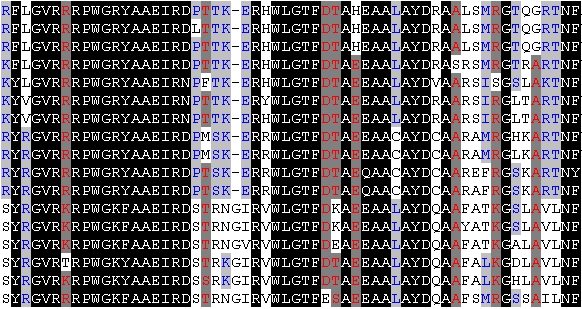


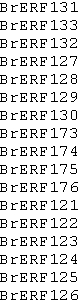


**ⅩI**

**Figure S1B**

Alignment of the AP2/ERF and additional domains of each ERF subfamily group (GroupⅤ~Ⅺ) from *B. rapa.* All groups had representative AP2 domains. Additionally, Group Ⅴhad consensus sequences [QMIEELL], Group Ⅵ had a conserved CRF domain, a TEH region and a putative MAP kinase phosphorylation site. Group Ⅵ-L had a conserved CRF domain and consensus sequences [FNxxxLxIP], [LPDxDFxD]. Group Ⅷ and Group Ⅹ had Ser rich region in N or C terminal ends. The black background represented the most conserved amino acid residues in each group. The black bar and arrows represented predicted α-helix and β-sheet regions, respectively, within the AP2/ERF domain. Asterisks represented amino acid residues that directly make contact with DNA (Allen et al., 1998).

**AP2 domain I**


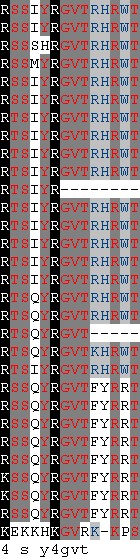

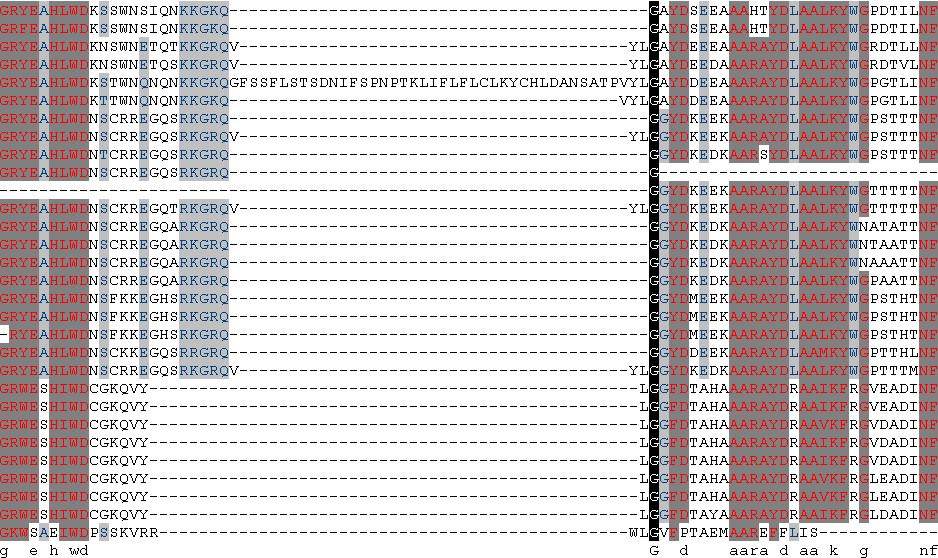


**AP2**

**Subfamily**


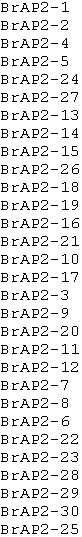

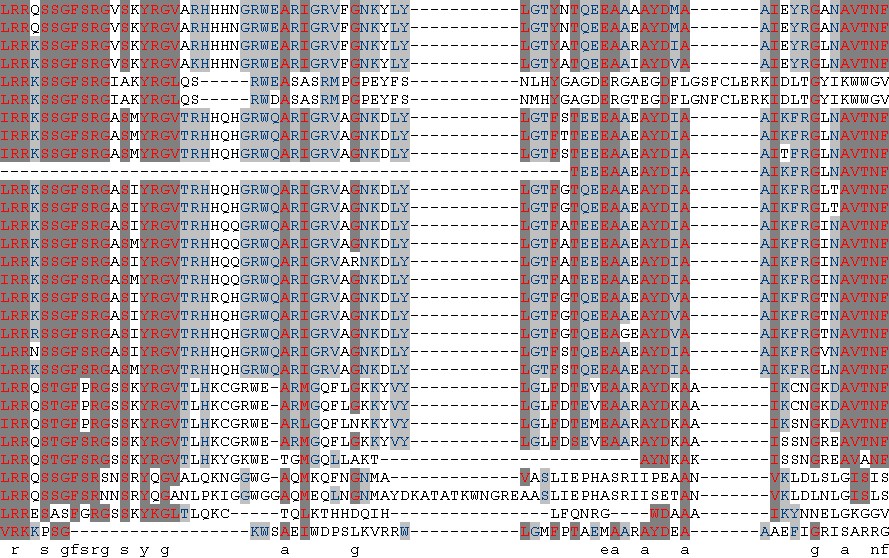


**AP2 domainⅡ**

**Figure S1C**

Alignment of the AP2/ERF domains of AP2 subfamily from *B. rapa.* The black background represented the most conserved amino acid residues in each group. Each AP2 subfamily member had two AP2/ERF domains, AP2 domain Ⅰand AP2 domain Ⅱ.


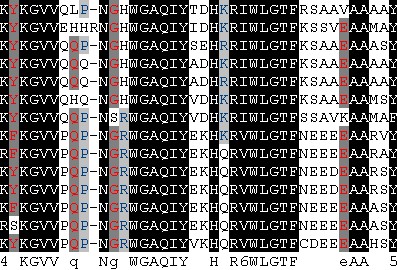

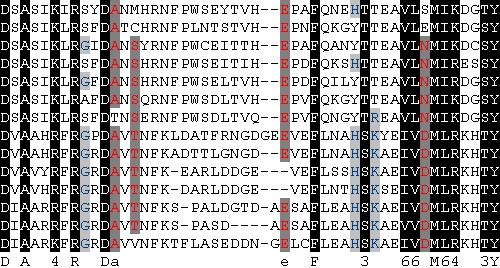


**AP2 domain**


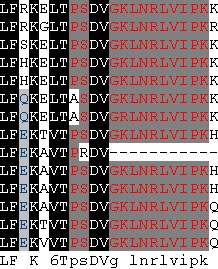

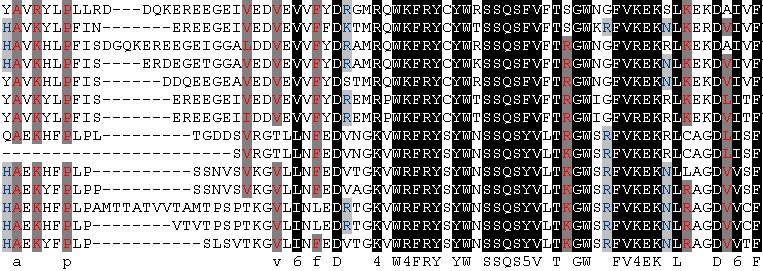


**B3 domain**

**Figure S1D**

Alignment of the AP2/ERF and B3 domains of RAV subfamily from *B. rapa.* The black background represented the most conserved amino acid residues in each group. Each RAV subfamily member had an AP2/ERF domain and a B3 domain.

**RAV**

**Subfamily**


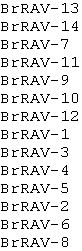

Supplement: Figure S1 — Alignment of the conserved amino acid sequences of each AP2/ERF subfamily group from B. rapa . (A) Alignment of the AP2/ERF domains and additional domains of each DREB subfamily group (GroupI∼IV) from B. rapa. (B) Alignment of the AP2/ERF and additional domains of each ERF subfamily group (GroupV∼XI) from B. rapa. (C) Alignment of the AP2/ERF domains of AP2 subfamily from B. rapa. (D) Alignment of the AP2/ERF and B3 domains of RAV subfamily from B. rapa. The black background represented the most conserved amino acid residues in each group. The black bar and arrows represented predicted α-helix and β-sheet regions, respectively, within the AP2/ERF domain. Asterisks represented amino acid residues that directly make contact with DNA. (DOC) [file pone.0083444.s001.doc]
