# Supplementary material for: Genome-Wide Identification, Phylogeny, Evolution and Expression Patterns of AP2/ERF Genes and Cytokinin Response Factors in Brassica rapa ssp. pekinensis
Source: PLoS One. 2013 Dec 30;8(12):e83444. doi: 10.1371/journal.pone.0083444 (PMC3875448; doi:10.1371/journal.pone.0083444)
Supplement: Figure S2 — Synteny analysis of BrCRFs in ±100kb region with score greater than 1500. Synteny analysis revealed evidence of the segmental duplication among BrCRFs. (DOC) [file pone.0083444.s002.doc]

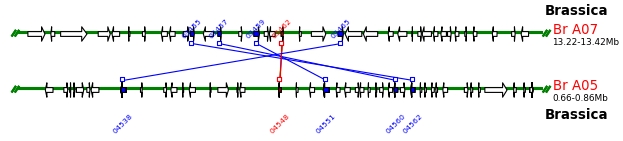


***BrCRF1***

***BrCRF2***


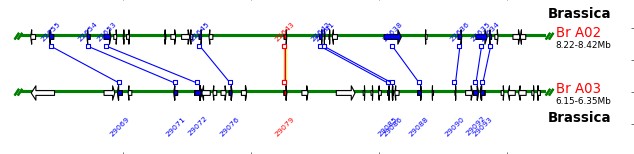


***BrCRF3***

***BrCRF5***


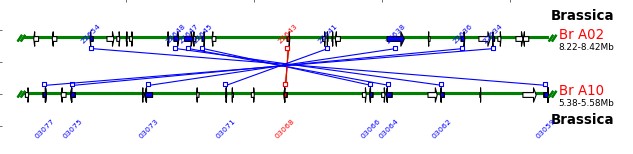


***BrCRF3***

***BrCRF4***


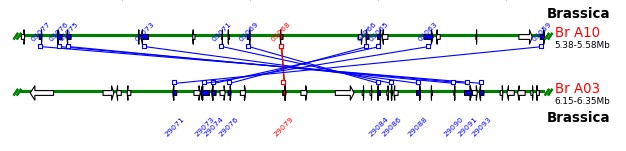


***BrCRF4***

***BrCRF5***


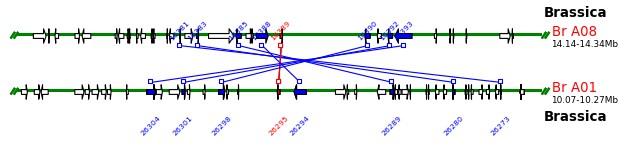


***BrCRF6***

***BrCRF8***


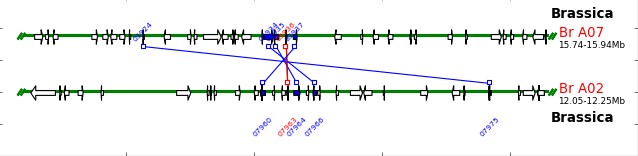


***BrCRF9***

***BrCRF10***


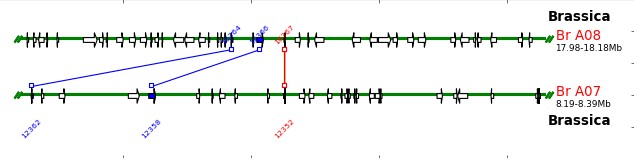


***BrCRF11***

***BrCRF12***


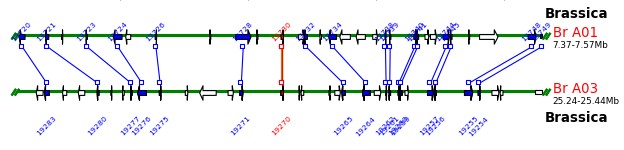


***BrCRF13***

***BrCRF14***


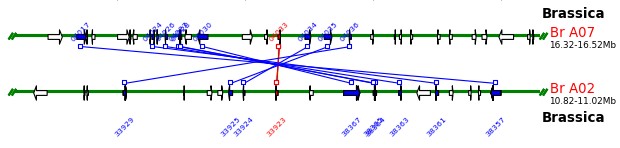


***BrCRF16***

***BrCRF18***


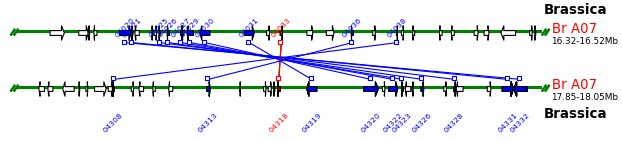


***BrCRF16***

***BrCRF17***


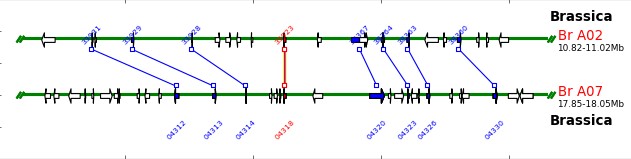


***BrCRF18***

***BrCRF17***


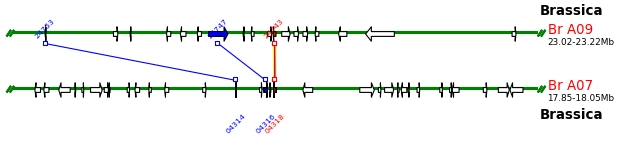


***BrCRF17***

***BrCRF21***


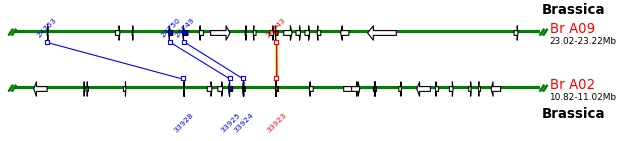


***BrCRF21***

***BrCRF18***


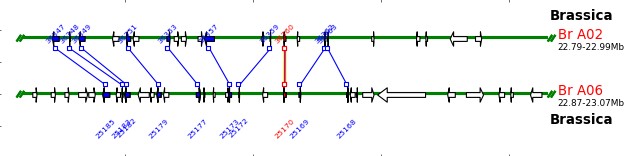


***BrCRF20***

***BrCRF19***

Figure S2. Synteny analysis of *BrCRFs* in ±100kb region with score greater than 1500. Synteny analysis revealed evidence of the segmental duplication among *BrCRFs*.
