# Supplementary material for: Genome-Wide Identification, Phylogeny, Evolution and Expression Patterns of AP2/ERF Genes and Cytokinin Response Factors in Brassica rapa ssp. pekinensis
Source: PLoS One. 2013 Dec 30;8(12):e83444. doi: 10.1371/journal.pone.0083444 (PMC3875448; doi:10.1371/journal.pone.0083444)
Supplement: Figure S3 — Neighbor joining tree of CRF proteins based on conserved CRF and AP2 domains of Br CRFs with their Arabidopsis counterparts. The tree could be divided into 5 Clades, Clade I, II, III, IV and V. (DOC) [file pone.0083444.s003.doc]

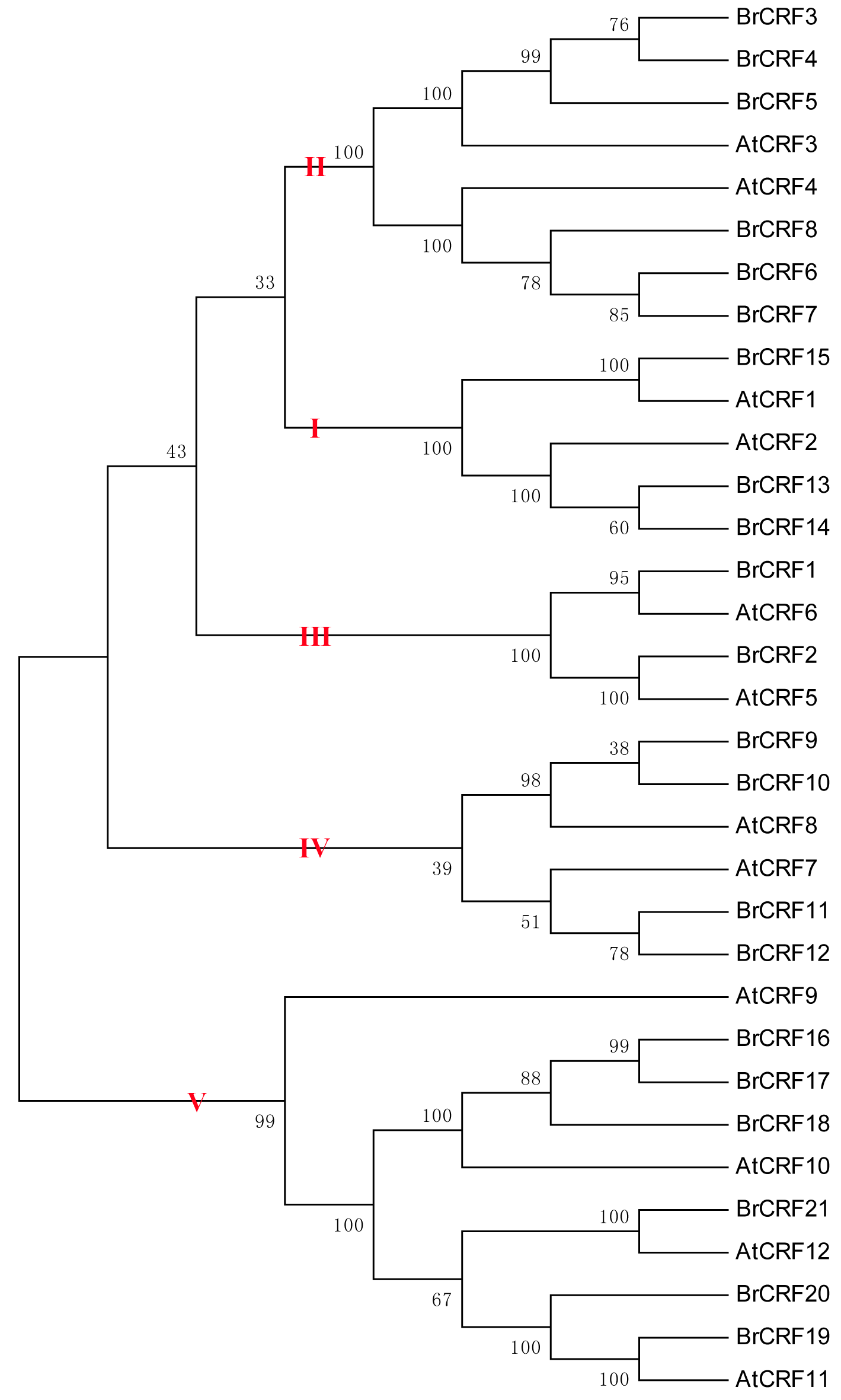


Figure S3. Neighbor joining tree of CRF proteins based on conserved CRF and AP2 domains of *Br*CRFs with their Arabidopsis counterparts. The tree could be divided into 5 Clades, Clade I, II, III, IV and V.
