# Supplementary material for: Genome-Wide Identification, Phylogeny, Evolution and Expression Patterns of AP2/ERF Genes and Cytokinin Response Factors in Brassica rapa ssp. pekinensis
Source: PLoS One. 2013 Dec 30;8(12):e83444. doi: 10.1371/journal.pone.0083444 (PMC3875448; doi:10.1371/journal.pone.0083444)
Supplement: Figure S4 — Neighbor joining tree of CRF proteins from 81 sequences identified by genus name and numerical identifiers from respective databases. The phylogenetic tree of CRF gene family contained A. thaliana (12), B. rapa (21), O. sativa (9), Z. mays (9), S. lycopersicum (12), P. trichocarpa (11) and P. patens (7). The tree could be divided into 3 groups, Group A, Group B and Group C. Besides, Group A could be further divided into Clade I, II and III, Group B could be further divided into Clade IV, VI, VII, VIII and IX. Group C contained a single Clade V. (DOC) [file pone.0083444.s004.doc]

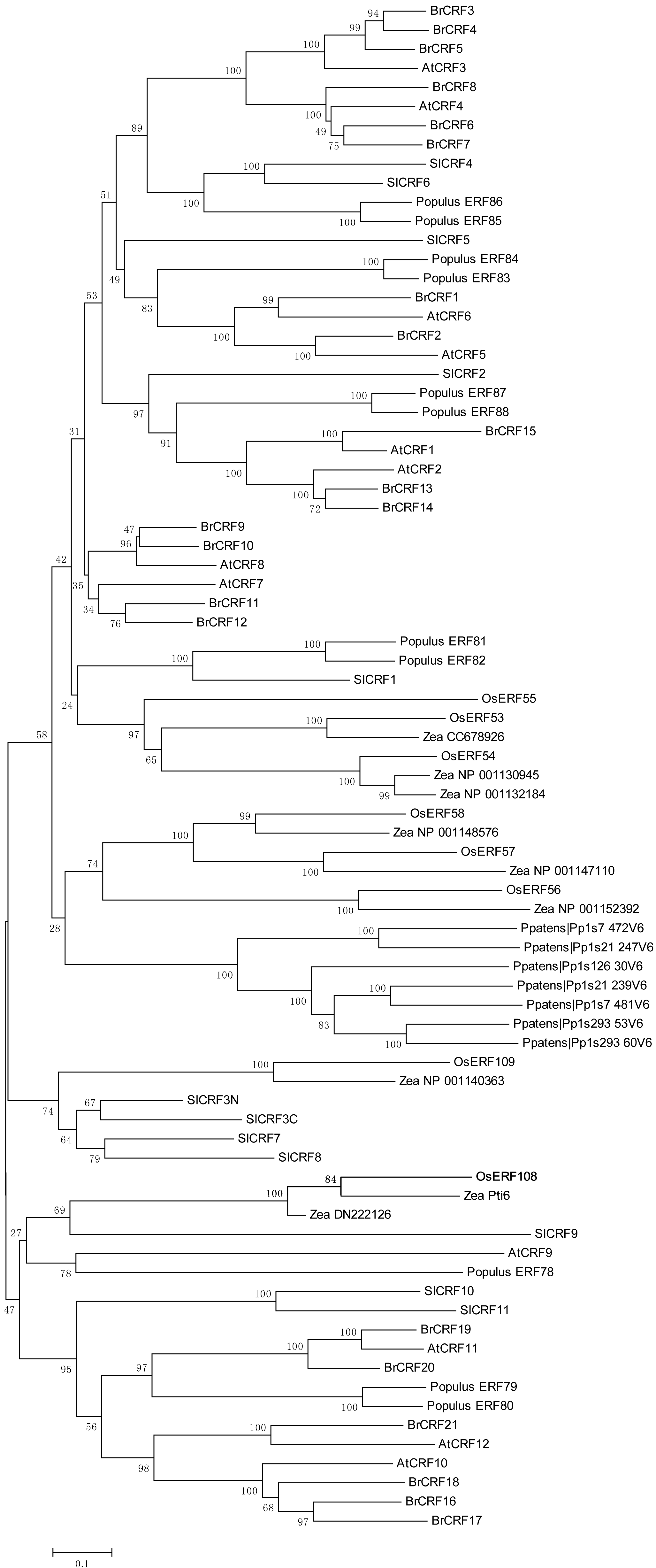


**Clade Ⅱ**

**Clade Ⅲ**

**Clade Ⅰ**

**Clade Ⅳ**

**Clade Ⅴ**

**Clade Ⅵ**

**Clade Ⅶ**

**Clade Ⅷ**

**Clade Ⅸ**

**Group A**

**Group C**

**Group B**

Figure S4. Neighbor joining tree of CRF proteins from 81 sequences identified by genus name and numerical identifiers from respective databases. The phylogenetic tree of CRF gene family contained *A.thaliana* (12), *B.rapa* (21), *O.sativa* (9), *Z.mays* (9), *S.lycopersicum* (12), *P.trichocarpa* (11) and *P.patens* (7). The tree could be divided into 3 groups, Group A, Group B and Group C. Besides, Group A could be further divided into Clade I, II and III, Group B could be further divided into Clade Ⅳ, Ⅵ, Ⅶ, Ⅷ and Ⅸ. Group C contained a single Clade Ⅴ.
