# Supplementary figures and images for: Genome-Wide Identification, Phylogeny, Evolution and Expression Patterns of AP2/ERF Genes and Cytokinin Response Factors in Brassica rapa ssp. pekinensis
Source: PLoS One. 2013 Dec 30;8(12):e83444. doi: 10.1371/journal.pone.0083444 (PMC3875448; doi:10.1371/journal.pone.0083444)

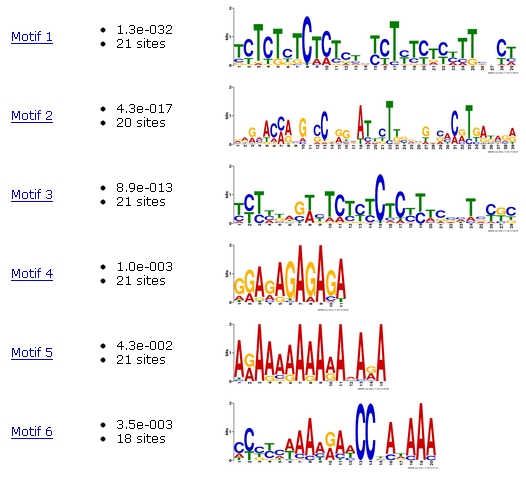


Figure S5. Motifs found with putative promoter regions of *BrCRFs* by MEME analysis.

Supplement: Figure S5 — Motifs found with putative promoter regions of BrCRFs by MEME analysis. (DOC) [file pone.0083444.s005.doc]
