# Supplementary material for: Genome-Wide Identification, Phylogeny, Evolution and Expression Patterns of AP2/ERF Genes and Cytokinin Response Factors in Brassica rapa ssp. pekinensis
Source: PLoS One. 2013 Dec 30;8(12):e83444. doi: 10.1371/journal.pone.0083444 (PMC3875448; doi:10.1371/journal.pone.0083444)
Supplement: Table S1 — Forward and reverse primers used in the qRT-PCR gene expression studies. (DOC) [file pone.0083444.s006.doc]

Table S1. Forward and reverse primers used in the qRT-PCR gene expression studies

| Primer name | Forward sequence | Reverse sequence |
| --- | --- | --- |
| *BrActin1* | CGCTTAACCCGAAAGCTAAC | TACGCCCACTAGCGTAAAG |
| *BrCRF1* | TACTCGGATTTGGTTAGGGACTT | CAGAGCGTTGTGACCTTTGAGAC |
| *BrCRF2* | GCGATTCGGATCAAAGGACG | AACGGAGAACAGAAGTAGGAGAACG |
| *BrCRF3* | CCGGAGTTTCCATGAAGG | AGAACGGTGACGGTTCGA |
| *BrCRF4* | ACCGTCGTCCACTCAAGG | TCGAAAGTACCAAGCCAAATC |
| *BrCRF5* | TATGTTGGACAGTACGTTTCTAG | TGAACCAATATCTTTAGTAAACTCA |
| *BrCRF6* | TCCGTTGGAGAAGATGTTAGT | ACATCAGGCCGTTGAAGA |
| *BrCRF7* | TCTTCGATCAGTCCATGTCTC | TCTCCACCTATGTTGCTGTTAT |
| *BrCRF8* | AACGCAGGAGGATTTGGC | CCGATACGCTGCTCTTGGA |
| *BrCRF9* | GAGGAGCCCTGCCGAGAAACA | TCGTCGGAGTCGGGAGGGTC |
| *BrCRF10* | AGAGGCGGACCAGCTTTCTGA | GATCTCCTTGACGAGGCGTTTT |
| *BrCRF11* | GTGGCTTGGGACGTTCGAGACG | CCAGCATCGGAACCTTCCTTCG |
| *BrCRF12* | AGCTCACGGTGGAATTCGC | CGGAATCTTGCTTCACAGCC |
| *BrCRF13* | ACCCAGACGCAACAGATTCCT | CCGACTTGACTACAACCGCTCT |
| *BrCRF14* | CAACAACCACCACCTTCAACATC | CCGGTACTAGCAGCACCAGAAT |
| *BrCRF15* | AATGGGCGGCGGAGATAA | AAACCGGCGAACCGAGAC |
| *BrCRF16* | CAGCCTCGCCAAGTGAAA | CTCTGCCTAACGCCAACA |
| *BrCRF17* | TGCCGAAGAGGGACAAAT | CGAACCCGAGCATAGTGA |
| *BrCRF18* | GTCCAGTGAAACGCATTGTC | CCTAATCTCAGCAGCCCAC |
| *BrCRF19* | GCTCCCTGTGATTCAACTGC | CTCGTCAATGAAGCAACCC |
| *BrCRF20* | GAGATCGACCGTCCTTTC | GGAGGTTTTGGCTTTGCT |
| *BrCRF21* | GACTCCTCTAGCGACGAATG | TGCCTCAGCCTAACTCCC |
