# Supplementary material for: Genome-Wide Identification, Phylogeny, Evolution and Expression Patterns of AP2/ERF Genes and Cytokinin Response Factors in Brassica rapa ssp. pekinensis
Source: PLoS One. 2013 Dec 30;8(12):e83444. doi: 10.1371/journal.pone.0083444 (PMC3875448; doi:10.1371/journal.pone.0083444)
Supplement: Table S4 — Ka and Ks values of CRF orthologs between B. rapa and A. thaliana . The BrCRFs and AtCRFs were both divided into 5 Clades based on the phylogenetic trees previously described. Values of Ka and Ks were determined. No orthologs of AtCRF9 were identified in B. rapa. Likewise, no orthologs of BrCRF7 existed in A. thaliana. (DOC) [file pone.0083444.s009.doc]

Table S4. *Ka* and *Ks* values of *CRF* orthologs between *B. rapa* and *A. thaliana***.** The *BrCRFs* and *AtCRFs* were both divided into 5 Clades based on the phylogenetic trees previously described. Values of *Ka* and *Ks* were determined**.** No orthologs of *AtCRF9* were identified in *B. rapa.* Likewise, no orthologs of *BrCRF7* existed in *A. thaliana.*

| Clades | *A. thaliana* | *B. rapa* | *Ka* value | *Ks* value |
| --- | --- | --- | --- | --- |
| Ⅰ | *AtCRF1* | *BrCRF15* | 0.17 | 0.53 |
|  | *AtCRF2* | *BrCRF13* | 0.12 | 0.62 |
|  |  | *BrCRF14* | 0.16 | 0.71 |
| Ⅱ | *AtCRF3* | *BrCRF3* | 0.17 | 0.55 |
|  |  | *BrCRF4* | 0.16 | 0.45 |
|  |  | *BrCRF5* | 0.19 | 0.57 |
|  | *AtCRF4* | *BrCRF6* | 0.15 | 0.44 |
|  |  | *BrCRF8* | 0.16 | 0.47 |
| Ⅲ | *AtCRF5* | *BrCRF2* | 0.19 | 0.91 |
|  | *AtCRF6* | *BrCRF1* | 0.26 | 0.89 |
| Ⅳ | *AtCRF7* | *BrCRF11* | 0.19 | 1.48 |
|  |  | *BrCRF12* | 0.15 | 1.19 |
|  | *AtCRF8* | *BrCRF9* | 0.12 | 0.60 |
|  |  | *BrCRF10* | 0.11 | 0.56 |
| Ⅴ | *AtCRF9* | — | — | — |
|  | *AtCRF10* | *BrCRF16* | 0.24 | 0.96 |
|  |  | *BrCRF17* | 0.25 | 0.76 |
|  |  | *BrCRF18* | 0.34 | 0.76 |
|  | *AtCRF11* | *BrCRF19* | 0.10 | 0.31 |
|  |  | *BrCRF20* | 0.17 | 0.37 |
|  | *AtCRF12* | *BrCRF21* | 0.27 | 0.51 |
