# Supplementary material for: Genome-Wide Identification, Phylogeny, Evolution and Expression Patterns of AP2/ERF Genes and Cytokinin Response Factors in Brassica rapa ssp. pekinensis
Source: PLoS One. 2013 Dec 30;8(12):e83444. doi: 10.1371/journal.pone.0083444 (PMC3875448; doi:10.1371/journal.pone.0083444)
Supplement: Table S5 — Analysis of the TEH sequence in N-terminal ends and Put MAPK Phos (Putative MAPK phosphorylation site) in C-terminal ends of several plant CRF family proteins. Proteins lacking both the TEH sequence and Put MAPK Phos were not listed. — represented no corresponding sequences existed. (DOC) [file pone.0083444.s010.doc]

Table S5. Analysis of the TEH sequence in N-terminal ends and Put MAPK Phos (Putative MAPK phosphorylation site) in C-terminal ends of several plant CRF family proteins. Proteins lacking both the TEH sequence and Put MAPK Phos were not listed. — represented no corresponding sequences existed.

| Species | Protein | TEH sequence | Put MAPK Phos |
| --- | --- | --- | --- |
| *Arabidopsis thaliana* | *At*CRF1 | TEK | SPVSVL |
|  | *At*CRF2 | TEH | SPVSVL |
|  | *At*CRF3 | TEH | SPTSVL |
|  | *At*CRF4 | TEH | SPTSVL |
|  | *At*CRF5 | TVH | SPTSVL |
|  | *At*CRF6 | TEN | SPTSVL |
| *Brassica rapa* | *Br*CRF1 | TEH | SPTSVL |
|  | *Br*CRF2 | TVH | SPTSVL |
|  | *Br*CRF3 | TEH | SPTSVL |
|  | *Br*CRF4 | TEH | SPTSVL |
|  | *Br*CRF5 | SEH | SPTSVL |
|  | *Br*CRF6 | TEH | SPTSVL |
|  | *Br*CRF7 | TEH | SPTSVL |
|  | *Br*CRF8 | TEH | SPTSVL |
|  | *Br*CRF13 | AEH | SPVSVL |
|  | *Br*CRF14 | TEH | SPVSVL |
|  | *Br*CRF15 | SEH | SPVSVL |
| *Solanum lycopersicum* | *Sl*CRF1 | SEH | SPTSVL |
|  | *Sl*CRF2 | TEH | SPKSVL |
|  | *Sl*CRF4 | TEH | SPTSVL |
|  | *Sl*CRF5 | DHH | SPTSVL |
|  | *Sl*CRF6 | TEH | SPTSVL |
|  | *Sl*CRF9 | — | SPSSVL |
|  | *Sl*CRF10 | — | SPASVF |
|  | *Sl*CRF11 | — | SSSSVL |
| *Populus trichocarpa* | *Populus*_ERF81 | TEH | SPISVL |
|  | *Populus*_ERF82 | TEH | SPTSVL |
|  | *Populus*_ERF83 | TEH | SPTSVL |
|  | *Populus*_ERF84 | TEH | SPTSVL |
|  | *Populus*_ERF85 | TEH | SPKSVL |
|  | *Populus*_ERF86 | TEH | SPKSVL |
|  | *Populus*_ERF87 | SEH | SPISVL |
|  | *Populus*_ERF88 | TEH | SPISVL |
|  | *Populus*_ERF78 | — | SPLSVL |
|  | *Populus*_ERF79 | — | SPASVL |
|  | *Populus*_ERF80 | — | SPASVL |
| *Oryza sativa* | *Os*ERF53 | TEH | SPTSVL |
|  | *Os*ERF54 | — | SPTSVL |
|  | *Os*ERF55 | — | SPVSVL |
|  | *Os*ERF56 | — | SPTSVL |
|  | *Os*ERF108 | — | SPSSVL |
| *Zea mays* | *Zea*_CC678926 | TEH | SPTSVL |
|  | *Zea*_NP_001130945 | — | SPTSVL |
|  | *Zea*_NP_001147717 | — | SPSSVL |
|  | *Zea*_NP_001132184 | — | SPTSVL |
|  | *Zea*_NP_001152392 | — | SPISVL |
| *Physcomitrella patens* | — | — | — |
