# Supplementary material for: Genome-Wide Identification, Phylogeny, Evolution and Expression Patterns of AP2/ERF Genes and Cytokinin Response Factors in Brassica rapa ssp. pekinensis
Source: PLoS One. 2013 Dec 30;8(12):e83444. doi: 10.1371/journal.pone.0083444 (PMC3875448; doi:10.1371/journal.pone.0083444)
Supplement: Table S6 — Summmary of abiotic-stress inducible cis -elements in the putative promoters of BrCRFs . Cis-elements with larger numbers were marked red. (DOC) [file pone.0083444.s011.doc]

Table S6. Summmary of abiotic-stress inducible *cis*-elements in the promoters of *BrCRFs*. *Cis*-elements with larger numbers were marked red.

| Abiotic  stress | Gene name  *cis*-element | ***BrCRF1*** | ***BrCRF2*** | ***BrCRF3*** | ***BrCRF4*** | ***BrCRF5*** | ***BrCRF6*** | ***BrCRF7*** | ***BrCRF8*** | ***BrCRF9*** | ***BrCRF10*** | ***BrCRF11*** |
| --- | --- | --- | --- | --- | --- | --- | --- | --- | --- | --- | --- | --- |
| Drought-stress | S000133 | 0 | 0 | 0 | 0 | 0 | 0 | 0 | 0 | 0 | 0 | 0 |
|  | S000153 | 2 | 0 | 1 | 0 | 1 | 1 | 0 | 1 | 1 | 2 | 1 |
|  | S000174 | 2 | 1 | 0 | 1 | 1 | 0 | 1 | 0 | 1 | 1 | 1 |
|  | S000175 | 0 | 0 | 0 | 0 | 0 | 0 | 0 | 0 | 0 | 0 | 0 |
|  | S000176 | 4 | 5 | 4 | 4 | 5 | 2 | 2 | 3 | 3 | 7 | 6 |
|  | S000177 | 2 | 1 | 0 | 3 | 0 | 0 | 0 | 1 | 0 | 0 | 2 |
|  | S000402 | 0 | 0 | 1 | 0 | 1 | 1 | 0 | 1 | 0 | 0 | 1 |
|  | S000408 | 1 | 3 | 2 | 0 | 2 | 2 | 5 | 1 | 3 | 5 | 0 |
|  | S000413 | 2 | 1 | 0 | 1 | 1 | 0 | 1 | 0 | 1 | 1 | 1 |
|  | S000414 | 4 | 4 | 6 | 2 | 4 | 4 | 4 | 7 | 0 | 0 | 0 |
|  | S000415 | 12 | 10 | 8 | 2 | 8 | 16 | 20 | 12 | 2 | 0 | 8 |
|  | S000418 | 0 | 0 | 1 | 0 | 1 | 1 | 0 | 1 | 0 | 1 | 1 |
| Salt-  stress | S000402 | 0 | 0 | 1 | 0 | 1 | 1 | 0 | 1 | 0 | 0 | 1 |
|  | S000418 | 0 | 0 | 1 | 0 | 1 | 1 | 0 | 1 | 0 | 1 | 1 |
|  | S000453 | 3 | 7 | 7 | 5 | 9 | 3 | 8 | 9 | 3 | 6 | 5 |
| Heat-  stress | S000030 | 4 | 1 | 1 | 0 | 2 | 2 | 6 | 2 | 4 | 6 | 4 |
|  | S000418 | 0 | 0 | 1 | 0 | 1 | 1 | 0 | 1 | 0 | 1 | 1 |
| Cold-  stress | S000153 | 2 | 0 | 1 | 0 | 1 | 1 | 0 | 1 | 1 | 2 | 1 |
|  | S000157 | 0 | 0 | 1 | 0 | 1 | 1 | 0 | 1 | 0 | 0 | 0 |
|  | S000402 | 0 | 0 | 1 | 0 | 1 | 1 | 0 | 1 | 0 | 0 | 1 |
|  | S000407 | 14 | 12 | 6 | 6 | 16 | 4 | 4 | 16 | 10 | 18 | 12 |
|  | S000418 | 0 | 0 | 1 | 0 | 1 | 1 | 0 | 1 | 0 | 1 | 1 |
| Wound-stress | S000159 | 0 | 0 | 0 | 0 | 0 | 0 | 0 | 0 | 0 | 0 | 0 |
|  | S000242 | 0 | 0 | 0 | 0 | 0 | 0 | 0 | 0 | 1 | 0 | 1 |
|  | S000244 | 1 | 0 | 0 | 0 | 1 | 0 | 1 | 0 | 0 | 0 | 0 |
|  | S000444 | 0 | 0 | 0 | 0 | 0 | 0 | 1 | 1 | 1 | 0 | 0 |
|  | S000457 | 9 | 11 | 6 | 5 | 3 | 7 | 6 | 4 | 5 | 7 | 4 |

| Abiotic  stress | Gene name  *cis*-element | ***BrCRF12*** | ***BrCRF13*** | ***BrCRF14*** | ***BrCRF15*** | ***BrCRF16*** | ***BrCRF17*** | ***BrCRF18*** | ***BrCRF19*** | ***BrCRF20*** | ***BrCRF21*** |
| --- | --- | --- | --- | --- | --- | --- | --- | --- | --- | --- | --- |
| Drought-stress | S000133 | 0 | 0 | 0 | 0 | 0 | 2 | 0 | 0 | 0 | 0 |
|  | S000153 | 1 | 0 | 0 | 0 | 2 | 1 | 1 | 1 | 1 | 1 |
|  | S000174 | 1 | 2 | 2 | 1 | 0 | 0 | 0 | 0 | 0 | 0 |
|  | S000175 | 1 | 0 | 0 | 0 | 0 | 0 | 2 | 0 | 0 | 0 |
|  | S000176 | 3 | 8 | 7 | 3 | 6 | 4 | 1 | 5 | 7 | 5 |
|  | S000177 | 0 | 2 | 2 | 0 | 3 | 1 | 0 | 2 | 4 | 0 |
|  | S000402 | 1 | 0 | 0 | 0 | 1 | 0 | 0 | 0 | 0 | 0 |
|  | S000408 | 11 | 1 | 3 | 4 | 6 | 5 | 6 | 3 | 0 | 4 |
|  | S000413 | 1 | 2 | 2 | 1 | 0 | 0 | 0 | 0 | 0 | 0 |
|  | S000414 | 0 | 8 | 4 | 4 | 0 | 2 | 1 | 3 | 2 | 0 |
|  | S000415 | 2 | 22 | 10 | 6 | 2 | 4 | 6 | 10 | 4 | 4 |
|  | S000418 | 1 | 0 | 0 | 0 | 2 | 1 | 1 | 0 | 0 | 0 |
| Salt-  stress | S000402 | 1 | 0 | 0 | 0 | 1 | 0 | 0 | 0 | 0 | 0 |
|  | S000418 | 1 | 0 | 0 | 0 | 2 | 1 | 1 | 0 | 0 | 4 |
|  | S000453 | 7 | 8 | 5 | 11 | 5 | 9 | 3 | 5 | 5 | 7 |
| Heat-  stress | S000030 | 5 | 1 | 6 | 4 | 3 | 1 | 1 | 5 | 1 | 6 |
|  | S000418 | 1 | 0 | 0 | 0 | 2 | 1 | 1 | 0 | 0 | 0 |
| Cold-  stress | S000153 | 1 | 0 | 0 | 0 | 2 | 1 | 1 | 1 | 1 | 1 |
|  | S000157 | 0 | 0 | 0 | 0 | 1 | 0 | 0 | 0 | 0 | 0 |
|  | S000402 | 1 | 0 | 0 | 0 | 1 | 0 | 0 | 0 | 0 | 0 |
|  | S000407 | 12 | 22 | 20 | 12 | 2 | 10 | 6 | 16 | 4 | 4 |
|  | S000418 | 1 | 0 | 0 | 0 | 2 | 1 | 1 | 0 | 0 | 0 |
| Wound-stress | S000159 | 0 | 0 | 0 | 1 | 0 | 2 | 0 | 0 | 0 | 0 |
|  | S000242 | 0 | 0 | 0 | 0 | 0 | 0 | 0 | 0 | 0 | 0 |
|  | S000244 | 0 | 0 | 0 | 0 | 0 | 0 | 0 | 0 | 0 | 0 |
|  | S000444 | 0 | 0 | 0 | 0 | 0 | 0 | 0 | 0 | 0 | 0 |
|  | S000457 | 7 | 9 | 7 | 5 | 7 | 5 | 5 | 8 | 5 | 5 |
