# Supplementary material for: Genome-Wide Identification, Phylogeny, Evolution and Expression Patterns of AP2/ERF Genes and Cytokinin Response Factors in Brassica rapa ssp. pekinensis
Source: PLoS One. 2013 Dec 30;8(12):e83444. doi: 10.1371/journal.pone.0083444 (PMC3875448; doi:10.1371/journal.pone.0083444)
Supplement: Table S7 — Gene ontology for motifs by GOMO analysis. BP stands for biological process, CC stands for cellular component and MF stands for molecular function. (DOC) [file pone.0083444.s012.doc]

Table S7. Gene ontology for motifs by GOMO analysis. BP stands for biological process, CC stands for cellular component and MF stands for molecular function.

Motif 1 TC-rich motif

| **GO term** | **GOMO score** | **Specificity** | **GO name** |
| --- | --- | --- | --- |
| [GO:0003700](http://amigo.geneontology.org/cgi-bin/amigo/term-details.cgi?term=GO:0003700) | 1.275e-13 | ~83% | | MF | transcription factor activity | | --- | --- | |
| [GO:0048481](http://amigo.geneontology.org/cgi-bin/amigo/term-details.cgi?term=GO:0048481) | 1.186e-02 | ~86% | | BP | ovule development | | --- | --- | |
| [GO:0009944](http://amigo.geneontology.org/cgi-bin/amigo/term-details.cgi?term=GO:0009944) | 1.321e-02 | 100% | | BP | polarity specification of adaxial/abaxial axis | | --- | --- | |
| [GO:0009860](http://amigo.geneontology.org/cgi-bin/amigo/term-details.cgi?term=GO:0009860) | 1.616e-02 | 100% | | BP | pollen tube growth | | --- | --- | |
| [GO:0005524](http://amigo.geneontology.org/cgi-bin/amigo/term-details.cgi?term=GO:0005524) | 1.636e-02 | 100% | | MF | ATP binding | | --- | --- | |
| [GO:0000148](http://amigo.geneontology.org/cgi-bin/amigo/term-details.cgi?term=GO:0000148) | 2.275e-02 | 100% | | CC | 1,3-beta-glucan synthase complex | | --- | --- | |
| [GO:0003843](http://amigo.geneontology.org/cgi-bin/amigo/term-details.cgi?term=GO:0003843) | 2.275e-02 | 100% | | MF | 1,3-beta-glucan synthase activity | | --- | --- | |
| [GO:0006075](http://amigo.geneontology.org/cgi-bin/amigo/term-details.cgi?term=GO:0006075) | 2.275e-02 | ~93% | | BP | 1,3-beta-glucan biosynthetic process | | --- | --- | |
| [GO:0009965](http://amigo.geneontology.org/cgi-bin/amigo/term-details.cgi?term=GO:0009965) | 2.429e-02 | ~52% | | BP | leaf morphogenesis | | --- | --- | |
| [GO:0010050](http://amigo.geneontology.org/cgi-bin/amigo/term-details.cgi?term=GO:0010050) | 2.434e-02 | 100% | | BP | vegetative phase change | | --- | --- | |
| [GO:0010599](http://amigo.geneontology.org/cgi-bin/amigo/term-details.cgi?term=GO:0010599) | 3.059e-02 | 100% | | BP | production of lsiRNA involved in RNA interference | | --- | --- | |
| [GO:0003777](http://amigo.geneontology.org/cgi-bin/amigo/term-details.cgi?term=GO:0003777) | 3.088e-02 | 80% | | MF | microtubule motor activity | | --- | --- | |
| [GO:0009524](http://amigo.geneontology.org/cgi-bin/amigo/term-details.cgi?term=GO:0009524) | 3.210e-02 | 100% | | CC | phragmoplast | | --- | --- | |
| [GO:0010051](http://amigo.geneontology.org/cgi-bin/amigo/term-details.cgi?term=GO:0010051) | 3.254e-02 | ~55% | | BP | xylem and phloem pattern formation | | --- | --- | |
| [GO:0004722](http://amigo.geneontology.org/cgi-bin/amigo/term-details.cgi?term=GO:0004722) | 3.936e-02 | ~54% | | MF | protein serine/threonine phosphatase activity | | --- | --- | |

Motif 2

| **GO term** | **GOMO score** | **Specificity** | **GO name** |
| --- | --- | --- | --- |
| [GO:0009570](http://amigo.geneontology.org/cgi-bin/amigo/term-details.cgi?term=GO:0009570) | 8.564e-03 | ~67% | | CC | chloroplast stroma | | --- | --- | |

Motif 3 TC-rich motif

| **GO term** | **GOMO score** | **Specificity** | **GO name** |
| --- | --- | --- | --- |
| [GO:0003700](http://amigo.geneontology.org/cgi-bin/amigo/term-details.cgi?term=GO:0003700) | 1.275e-13 | ~83% | | MF | transcription factor activity | | --- | --- | |
| [GO:0048481](http://amigo.geneontology.org/cgi-bin/amigo/term-details.cgi?term=GO:0048481) | 1.186e-02 | ~86% | | BP | ovule development | | --- | --- | |
| [GO:0005524](http://amigo.geneontology.org/cgi-bin/amigo/term-details.cgi?term=GO:0005524) | 1.636e-02 | 100% | | MF | ATP binding | | --- | --- | |
| [GO:0009944](http://amigo.geneontology.org/cgi-bin/amigo/term-details.cgi?term=GO:0009944) | 1.321e-02 | 100% | | BP | polarity specification of adaxial/abaxial axis | | --- | --- | |
| [GO:0003777](http://amigo.geneontology.org/cgi-bin/amigo/term-details.cgi?term=GO:0003777) | 3.088e-02 | 80% | | MF | microtubule motor activity | | --- | --- | |
| [GO:0009738](http://amigo.geneontology.org/cgi-bin/amigo/term-details.cgi?term=GO:0009738) | 3.436e-04 | 100% | | BP | abscisic acid mediated signaling pathway | | --- | --- | |
| [GO:0035196](http://amigo.geneontology.org/cgi-bin/amigo/term-details.cgi?term=GO:0035196) | 2.403e-02 | ~95% | | BP | production of miRNAs involved in gene silencing by miRNA | | --- | --- | |

Motif 4 AG-rich motif

| **GO term** | **GOMO score** | **Specificity** | | **GO name** | |
| --- | --- | --- | --- | --- | --- |
| [GO:0003700](http://amigo.geneontology.org/cgi-bin/amigo/term-details.cgi?term=GO:0003700) | 1.388e-14 | | ~83% | | | MF | transcription factor activity | | --- | --- | |
| [GO:0010152](http://amigo.geneontology.org/cgi-bin/amigo/term-details.cgi?term=GO:0010152) | 5.184e-03 | | 100% | | | BP | pollen maturation | | --- | --- | |
| [GO:0005516](http://amigo.geneontology.org/cgi-bin/amigo/term-details.cgi?term=GO:0005516) | 1.522e-02 | | 100% | | | MF | calmodulin binding | | --- | --- | |
| [GO:0005524](http://amigo.geneontology.org/cgi-bin/amigo/term-details.cgi?term=GO:0005524) | 1.743e-02 | | 100% | | | MF | ATP binding | | --- | --- | |
| [GO:0009753](http://amigo.geneontology.org/cgi-bin/amigo/term-details.cgi?term=GO:0009753) | 1.804e-02 | | ~45% | | | BP | response to jasmonic acid stimulus | | --- | --- | |
| [GO:0009735](http://amigo.geneontology.org/cgi-bin/amigo/term-details.cgi?term=GO:0009735) | 1.831e-02 | | ~67% | | | BP | response to cytokinin stimulus | | --- | --- | |
| [GO:0009744](http://amigo.geneontology.org/cgi-bin/amigo/term-details.cgi?term=GO:0009744) | 1.876e-04 | | ~67% | | | BP | response to sucrose stimulus | | --- | --- | |
| [GO:0009651](http://amigo.geneontology.org/cgi-bin/amigo/term-details.cgi?term=GO:0009651) | 2.165e-02 | | ~38% | | | BP | response to salt stress | | --- | --- | |
| [GO:0009965](http://amigo.geneontology.org/cgi-bin/amigo/term-details.cgi?term=GO:0009965) | 2.253e-02 | | ~52% | | | BP | leaf morphogenesis | | --- | --- | |
| [GO:0003777](http://amigo.geneontology.org/cgi-bin/amigo/term-details.cgi?term=GO:0003777) | 2.489e-02 | | 80% | | | MF | microtubule motor activity | | --- | --- | |
| [GO:0010048](http://amigo.geneontology.org/cgi-bin/amigo/term-details.cgi?term=GO:0010048) | 2.260e-02 | | 100% | | | BP | vernalization response | | --- | --- | |
| [GO:0009751](http://amigo.geneontology.org/cgi-bin/amigo/term-details.cgi?term=GO:0009751) | 2.773e-02 | | 50% | | | BP | response to salicylic acid stimulus | | --- | --- | |
| [GO:0009733](http://amigo.geneontology.org/cgi-bin/amigo/term-details.cgi?term=GO:0009733) | 3.036e-02 | | ~43% | | | BP | response to auxin stimulus | | --- | --- | |
| [GO:0010051](http://amigo.geneontology.org/cgi-bin/amigo/term-details.cgi?term=GO:0010051) | 3.154e-02 | | ~55% | | | BP | xylem and phloem pattern formation | | --- | --- | |

Motif 5 AG-rich motif

| **GO term** | **GOMO score** | **Specificity** | **GO name** |
| --- | --- | --- | --- |
| [GO:0003700](http://amigo.geneontology.org/cgi-bin/amigo/term-details.cgi?term=GO:0003700) | 2.703e-09 | ~83% | | MF | transcription factor activity | | --- | --- | |
| [GO:0009736](http://amigo.geneontology.org/cgi-bin/amigo/term-details.cgi?term=GO:0009736) | 1.717e-02 | 100% | | BP | cytokinin mediated signaling pathway | | --- | --- | |

Motif 6 No significant GO-term could be associated with this motif.
