# Supplementary material for: Genome-Wide Identification, Phylogeny, Evolution and Expression Patterns of AP2/ERF Genes and Cytokinin Response Factors in Brassica rapa ssp. pekinensis
Source: PLoS One. 2013 Dec 30;8(12):e83444. doi: 10.1371/journal.pone.0083444 (PMC3875448; doi:10.1371/journal.pone.0083444)
Supplement: Table S8 — Summary of AP2/ERF family genes whose biological functions have been reported. (DOC) [file pone.0083444.s013.doc]

Table S8. Summary of AP2/ERF family genes whose biological functions have been reported.

| **Genes** | **Functions** | **Species** | **References** |
| --- | --- | --- | --- |
| *ANT* | Ovule and female gametophyte development | Arabidopsis |  |
| *TINY* | Growth regulation | Arabidopsis |  |
| *CBF1-4,DREB1A-D* | Freezing and dehydration tolerance | Arabidopsis |  |
| *ABI4* | ABA response and sugar signaling | Arabidopsis |  |
| *LEP* | Leaf petiole development | Arabidopsis |  |
| *ERF1* | Disease resistance | Arabidopsis |  |
| *ESR1/DRN* | Organ identity | Arabidopsis |  |
| *WIN1/SHNs* | Wax accumulation | Arabidopsis |  |
| *AtERF4* | Ethylene, JA, and ABA response | Arabidopsis |  |
| *AtERF7* | ABA response | Arabidopsis |  |
| *ABR1* | ABA response | Arabidopsis |  |
| *Bolita* | Cell expansion and proliferation | Arabidopsis |  |
| *OsDREB1A, B* | High salinity and freezing | Arabidopsis |  |
| *DREB2A* | Drought and freezing tolerance | Arabidopsis |  |
| *LpCBF3* | Freezing tolerance | Arabidopsis |  |
| *WXP1, WXP2* | Wax accumulation | Arabidopsis |  |
| *AtERF14* | Biotic stress | Arabidopsis |  |
| *DREB2C* | Thermotolerance | Arabidopsis |  |
| *GmDREB2* | High salt and drought | Arabidopsis |  |
| *JcERF* | Salt and freezing tolerance | Arabidopsis |  |
| *AtERF38* | Secondary wall metabolism | Arabidopsis |  |
| *GmSGR* | Reduced ABA-sensitivity, enhanced salt sensitivity | Arabidopsis |  |
| *ORA59* | Essential integrator of JA and ethylene signal transduction | Arabidopsis |  |
| *OsERF1* | Growth and development | Arabidopsis | 30] |
| *OsDREB1F* | Salt, drought and low temperature tolerance | Arabidopsis, Rice |  |
| *AtCRF2* | Increase the chloroplast division rate | Arabidopsis |  |
| *RAP2.2* | Low oxygen response | Arabidopsis |  |
| *AtCRF5* | Pathogen resistance | Arabidopsis |  |
| *RAP2.6* | ABA, salt and osmotic tolerance | Arabidopsis |  |
| *AtERF73/HRE1* | Modulating ethylene response | Arabidopsis |  |
| *WIND1* | Cell dedifferentiation | Arabidopsis |  |
| *HARDY* | Drought and salt tolerance | Arabidopsis |  |
| *AtERF71/HRE2,* | Osmotic tolerance | Arabidopsis |  |
| *RAP2.11* | Response to low-potassium conditions | Arabidopsis |  |
| *AtCRF4* | Cold tolerance | Arabidopsis | Compton MA, unpublished Master thesis |
| *AtCRF5* | Hormonal crosstalk and sugar metabolism | Arabidopsis | Ketelsen B, unpublished PhD thesis |
| *BrERF4* | Cell expansion restriction | Arabidopsis |  |
| *MsERF11* | Salt tolerance | Arabidopsis |  |
| *OsAP21* | Drought and salt tolerance | Arabidopsis |  |
| *FZP* | Floral meristem identity | Rice |  |
| *Sub1* | Submergence tolerance | Rice |  |
| *TERF1* | Drought and high salt tolerance | Rice |  |
| *TSRF1* | Drought tolerance | Rice |  |
| *OsERF3* | Herbivore-induced tolerance | Rice |  |
| OsEATB | Internode elongation restriction | Rice |  |
| *TERF2* | Cold tolerance | Rice |  |
| *OsDREB2A* | Dehydration and salt tolerance | Rice |  |
| *OsWR1* | Drought tolerance | Rice |  |
| *Pti4, Pti6* | Disease resistance | Tomato |  |
| *Pti5* | Disease resistance | Tomato |  |
| *JERF3* | Salt tolerance | Tomato |  |
| *TERF1* | Salt tolerance | Tomato |  |
| *ERF2* | Seed germination | Tomato |  |
| *LeERF3b* | Drought, dessication and low temp response | Tomato |  |
| *SlERF1* | Salt tolerance | Tomato |  |
| *SlERF5* | Drought and cold tolerance | Tomato |  |
| *SlERF6* | Ripening and carotenoid accumulation | Tomato |  |
| *SlERF4* | Suppress ethylene responses | Tomato |  |
| *SlDREB* | Leaf expansion and internode elongation | Tomato |  |
| *Tsi1* | Salt tolerance, disease resistance | Tobacco |  |
| *NtERF5* | Disease resistance | Tobacco |  |
| *OPBP1* | Salt tolerance, disease resistance | Tobacco |  |
| *SodERF3* | Salt and drought tolerance | Tobacco |  |
| *TiERF1* | Biotic stress | Tobacco |  |
| *MsERF8* | Salt tolerance | Tobacco |  |
| *OjERF* | Drought tolerance | Tobacco |  |
| *GmERF7* | Salt tolerance | Tobacco |  |
| *BrERF11* | Disease resistance | Tobacco |  |
| *TSRF1* | ABA response | Tobacco |  |
| *CBF like* | Cold tolerance | Rape |  |
| *BBM* | Promoting cell proliferation and morphogenesis during embryogenesis | Rape |  |
| *BNCBF5,BNCBF17* | Freezing tolerance | Rape |  |
| *BBM1* | Somatic embryogenesis and embryo development | Rape |  |
| *BnaERF-B3-hy15* | Freezing tolerance, ABA response | Rape |  |

1. Klucher KM, Chow H, Reiser L, Fischer RL (1996) The AINTEGUMENTA gene of arabidopsis required for ovule and female gametophyte development is related to the floral homeotic gene APETALA2. Plant Cell 8: 137-153.

2. Wilson K, Long D, Swinburne J, Coupland G (1996) A dissociation insertion causes a semidominant mutation that increases expression of *TINY*, an arabidopsis gene related to APETALA2. Plant Cell 8: 659-671.

3. Liu Q, Kasuga M, Sakuma Y, Abe H, Miura S, et al. (1998) Two transcription factors, *DREB1* and *DREB2*, with an EREBP/AP2 DNA binding domain separate two cellular signal transduction pathways in drought- and low-temperature-responsive gene expression, respectively, in *Arabidopsis*. Plant Cell 10: 1391-1406.

4. Gilmour SJ, Sebolt AM, Salazar MP, Everard JD, Thomashow MF (2000) Overexpression of the Arabidopsis *CBF3* transcriptional activator mimics multiple biochemical changes associated with cold acclimation. Plant Physiology 124: 1854-1865.

5. Haake V, Cook D, Riechmann JL, Pineda O, Thomashow MF, et al. (2002) Transcription factor *CBF4* is a regulator of drought adaptation in *Arabidopsis*. Plant Physiology 130: 639-648.

6. Finkelstein RR, Wang ML, Lynch TJ, Rao S, Goodman HM (1998) The *Arabidopsis* abscisic acid response locus *ABI4* encodes an APETALA2 domain protein. Plant Cell 10: 1043-1054.

7. Arenas-Huertero F, Arroyo A, Zhou L, Sheen J, Leon P (2000) Analysis of Arabidopsis glucose insensitive mutants, *gin5* and *gin6*, reveals a central role of the plant hormone ABA in the regulation of plant vegetative development by sugar. Genes & Development 14: 2085-2096.

8. Huijser C, Kortstee A, Pego J, Weisbeek P, Wisman E, et al. (2000) The *Arabidopsis* SUCROSE UNCOUPLED-6 gene is identical to ABSCISIC ACID INSENSITIVE-4: involvement of abscisic acid in sugar responses. Plant Journal 23: 577-585.

9. van der Graaff E, Den Dulk-Ras A, Hooykaas PJJ, Keller B (2000) Activation tagging of the LEAFY PETIOLE gene affects leaf petiole development in *Arabidopsis thaliana*. Development 127: 4971-4980.

10. Solano R, Stepanova A, Chao QM, Ecker JR (1998) Nuclear events in ethylene signaling: a transcriptional cascade mediated by *ETHYLENE-INSENSITIVE3* and *ETHYLENE-RESPONSE-FACTOR1*. Genes & Development 12: 3703-3714.

11. Berrocal-Lobo M, Molina A, Solano R (2002) Constitutive expression of *ETHYLENE-RESPONSE-FACTOR1* in *Arabidopsis* confers resistance to several necrotrophic fungi. Plant Journal 29: 23-32.

12. Banno H, Chua NH (2002) Overexpression of Arabidopsis *ESR1* induces initiation of shoot regeneration. Plant and Cell Physiology 43: S39-S39.

13. Kirch T, Simon R, Grunewald M, Werr W (2003) The DORNROSCHEN/ENHANCER OF SHOOT REGENERATION1 gene of Arabidopsis acts in the control of meristem cell fate and lateral organ development. Plant Cell 15: 694-705.

14. Aharoni A, Dixit S, Jetter R, Thoenes E, van Arkel G, et al. (2004) The SHINE clade of AP2 domain transcription factors activates wax biosynthesis, alters cuticle properties, and confers drought tolerance when overexpressed in *Arabidopsis*. Plant Cell 16: 2463-2480.

15. Broun P, Poindexter P, Osborne E, Jiang CZ, Riechmann JL (2004) *WIN1*, a transcriptional activator of epidermal wax accumulation in *Arabidopsis*. Proceedings of the National Academy of Sciences of the United States of America 101: 4706-4711.

16. Song CP, Agarwal M, Ohta M, Guo Y, Halfter U, et al. (2005) Role of an *Arabidopsis* AP2/EREBP-type transcriptional repressor in abscisic acid and drought stress responses. Plant Cell 17: 2384-2396.

17. Pandey GK, Grant JJ, Cheong YH, Kim BG, Li LG, et al. (2005) *ABR1*, an APETALA2-domain transcription factor that functions as a repressor of ABA response in *Arabidopsis*. Plant Physiology 139: 1185-1193.

18. Marsch-Martinez N, Greco R, Becker JD, Dixit S, Bergervoet JHW, et al. (2006) *BOLITA*, an Arabidopsis AP2/ERF-like transcription factor that affects cell expansion and proliferation/differentiation pathways. Plant Molecular Biology 62: 825-843.

19. Ito Y, Katsura K, Maruyama K, Taji T, Kobayashi M, et al. (2006) Functional analysis of rice DREB1/CBF-type transcription factors involved in cold-responsive gene expression in transgenic rice. Plant and Cell Physiology 47: 141-153.

20. Sakuma Y, Maruyama K, Osakabe Y, Qin F, Seki M, et al. (2006) Functional analysis of an Arabidopsis transcription factor, *DREB2A*, involved in drought-responsive gene expression. Plant Cell 18: 1292-1309.

21. Xiong YW, Fei SZ (2006) Functional and phylogenetic analysis of a DREB/CBF-like gene in perennial ryegrass (*Lolium perenne L.*). Planta 224: 878-888.

22. Zhang JY, Broeckling CD, Sumner LW, Wang ZY (2007) Heterologous expression of two *Medicago truncatula* putative ERF transcription factor genes, *WXP1* and *WXP2*, in Arabidopsis led to increased leaf wax accumulation and improved drought tolerance, but differential response in freezing tolerance. Plant Molecular Biology 64: 265-278.

23. Onate-Sanchez L, Anderson JP, Young J, Singh KB (2007) *AtERF14*, a member of the ERF family of transcription factors, plays a nonredundant role in plant defense. Plant Physiology 143: 400-409.

24. Lim CJ, Hwang JE, Chen H, Hong JK, Yang KA, et al. (2007) Over-expression of the Arabidopsis DRE/CRT-binding transcription factor *DREB2C* enhances thermotolerance. Biochemical and Biophysical Research Communications 362: 431-436.

25. Chen M, Wang QY, Cheng XG, Xu ZS, Li LC, et al. (2007) *GmDREB2*, a soybean DRE-binding transcription factor, conferred drought and high-salt tolerance in transgenic plants. Biochemical and Biophysical Research Communications 353: 299-305.

26. Tang MJ, Sun JW, Liu Y, Chen F, Shen SH (2007) Isolation and functional characterization of the JcERF gene, a putative AP2/EREBP domain-containing transcription factor, in the woody oil plant Jatropha curcas. Plant Molecular Biology 63: 419-428.

27. Lasserre E, Jobet E, Llauro C, Delseny M (2008) *AtERF38* (*At2g35700*), an AP2/ERF family transcription factor gene from Arabidopsis thaliana, is expressed in specific cell types of roots, stems and seeds that undergo suberization. Plant Physiology and Biochemistry 46: 1051-1061.

28. Wang CM, Wang HW, Zhang JS, Chen SY (2008) A seed-specific AP2-domain transcription factor from soybean plays a certain role in regulation of seed germination. Science in China Series C-Life Sciences 51: 336-345.

29. Pre M, Atallah M, Champion A, De Vos M, Pieterse CMJ, et al. (2008) The AP2/ERF domain transcription factor *ORA59* integrates jasmonic acid and ethylene signals in plant defense. Plant Physiology 147: 1347-1357.

30. Hu YB, Zhao LF, Chong K, Wang T (2008) Overexpression of *OsERF1*, a novel rice ERF gene, up-regulates ethylene-responsive genes expression besides affects growth and development in *Arabidopsis*. Journal of plant physiology 165: 1717-1725.

31. Wang QY, Guan YC, Wu YR, Chen HL, Chen F, et al. (2008) Overexpression of a rice OsDREB1F gene increases salt, drought, and low temperature tolerance in both Arabidopsis and rice. Plant Molecular Biology 67: 589-602.

32. Okazaki K, Kabeya Y, Suzuki K, Mori T, Ichikawa T, et al. (2009) The PLASTID DIVISION1 and 2 Components of the Chloroplast Division Machinery Determine the Rate of Chloroplast Division in Land Plant Cell Differentiation. Plant Cell 21: 1769-1780.

33. Hinz M, Wilson IW, Yang J, Buerstenbinder K, Llewellyn D, et al. (2010) Arabidopsis *RAP2.2*: An Ethylene Response Transcription Factor That Is Important for Hypoxia Survival. Plant Physiology 153: 757-772.

34. Liang YS, Ermawati N, Cha JY, Jung MH, Su'udi M, et al. (2010) Overexpression of an AP2/ERF-type Transcription Factor *CRF5* Confers Pathogen Resistance to Arabidopsis Plants. Journal of the Korean Society for Applied Biological Chemistry 53: 142-148.

35. Zhu Q, Zhang JT, Gao XS, Tong JH, Xiao LT, et al. (2010) The Arabidopsis AP2/ERF transcription factor *RAP2.6* participates in ABA, salt and osmotic stress responses. Gene 457: 1-12.

36. Yang CY, Hsu FC, Li JP, Wang NN, Shih MC (2011) The AP2/ERF Transcription Factor *AtERF73/HRE1* Modulates Ethylene Responses during Hypoxia in *Arabidopsis*. Plant Physiology 156: 202-212.

37. Iwase A, Mitsuda N, Koyama T, Hiratsu K, Kojima M, et al. (2011) The AP2/ERF Transcription Factor *WIND1* Controls Cell Dedifferentiation in *Arabidopsis*. Current Biology 21: 508-514.

38. Abogadallah GM, Nada RM, Malinowski R, Quick P (2011) Overexpression of *HARDY*, an AP2/ERF gene from Arabidopsis, improves drought and salt tolerance by reducing transpiration and sodium uptake in transgenic *Trifolium alexandrinum L*. Planta 233: 1265-1276.

39. Park HY, Seok HY, Woo DH, Lee SY, Tarte VN, et al. (2011) *AtERF71/HRE2* transcription factor mediates osmotic stress response as well as hypoxia response in *Arabidopsis*. Biochemical and Biophysical Research Communications 414: 135-141.

40. Kim MJ, Ruzicka D, Shin R, Schachtman DP (2012) The Arabidopsis AP2/ERF Transcription Factor *RAP2.11* Modulates Plant Response to Low-Potassium Conditions. Molecular Plant 5: 1042-1057.

41. Park JB, Sendon PM, Kwon SH, Seo HS, Park SK, et al. (2012) Overexpression of Stress-Related Genes, *BrERF4* and *AtMYB44*, in *Arabidopsis thaliana* Alters Cell Expansion but Not Cell Proliferation During Leaf Growth. Journal of Plant Biology 55: 406-412.

42. Chen TT, Yang QC, Gruber M, Kang JM, Sun Y, et al. (2012) Expression of an alfalfa (*Medicago sativa L*.) ethylene response factor gene *MsERF8* in tobacco plants enhances resistance to salinity. Molecular Biology Reports 39: 6067-6075.

43. Jin XF, Xue Y, Wang R, Xu RR, Bian L, et al. (2013) Transcription factor OsAP21 gene increases salt/drought tolerance in transgenic *Arabidopsis thaliana*. Molecular Biology Reports 40: 1743-1752.

44. Komatsu M, Chujo A, Nagato Y, Shimamoto K, Kyozuka J (2003) FRIZZY PANICLE is required to prevent the formation of axillary meristems and to establish floral meristem identity in rice spikelets. Development 130: 3841-3850.

45. Xu K, Xu X, Fukao T, Canlas P, Maghirang-Rodriguez R, et al. (2006) *Sub1A* is an ethylene-response-factor-like gene that confers submergence tolerance to rice. Nature 442: 705-708.

46. Gao SM, Zhang HW, Tian Y, Li F, Zhang ZJ, et al. (2008) Expression of *TERF1* in rice regulates expression of stress-responsive genes and enhances tolerance to drought and high-salinity. Plant cell reports 27: 1787-1795.

47. Quan RD, Hu SJ, Zhang ZL, Zhang HW, Zhang ZJ, et al. (2010) Overexpression of an ERF transcription factor *TSRF1* improves rice drought tolerance. Plant Biotechnology Journal 8: 476-488.

48. Lu J, Ju HP, Zhou GX, Zhu CS, Erb M, et al. (2011) An EAR-motif-containing ERF transcription factor affects herbivore-induced signaling, defense and resistance in rice. Plant Journal 68: 583-596.

49. Qi WW, Sun F, Wang QJ, Chen ML, Huang YQ, et al. (2011) Rice Ethylene-Response AP2/ERF Factor *OsEATB* Restricts Internode Elongation by Down-Regulating a Gibberellin Biosynthetic Gene. Plant Physiology 157: 216-228.

50. Tian Y, Zhang HW, Pan XW, Chen XL, Zhang ZJ, et al. (2011) Overexpression of ethylene response factor *TERF2* confers cold tolerance in rice seedlings. Transgenic Research 20: 857-866.

51. Mallikarjuna G, Mallikarjuna K, Reddy MK, Kaul T (2011) Expression of *OsDREB2A* transcription factor confers enhanced dehydration and salt stress tolerance in rice (*Oryza sativa L*.). Biotechnology Letters 33: 1689-1697.

52. Wang YH, Wan LY, Zhang LX, Zhang ZJ, Zhang HW, et al. (2012) An ethylene response factor *OsWR1* responsive to drought stress transcriptionally activates wax synthesis related genes and increases wax production in rice. Plant Molecular Biology 78: 275-288.

53. Zhou JM, Tang XY, Martin GB (1997) The Pto kinase conferring resistance to tomato bacterial speck disease interacts with proteins that bind a *cis*-element of pathogenesis-related genes. Embo Journal 16: 3207-3218.

54. Gu YQ, Wildermuth MC, Chakravarthy S, Loh YT, Yang CM, et al. (2002) Tomato transcription factors *Pti4, Pti5*, and *Pti6* activate defense responses when expressed in *Arabidopsis*. Plant Cell 14: 817-831.

55. He P, Warren RF, Zhao TH, Shan LB, Zhu LH, et al. (2001) Overexpression of *Pti5* in tomato potentiates pathogen-induced defense gene expression and enhances disease resistance to Pseudomonas syringae pv. tomato. Molecular Plant-Microbe Interactions 14: 1453-1457.

56. Wang H, Huang ZJ, Chen Q, Zhang ZJ, Zhang HB, et al. (2004) Ectopic overexpression of tomato *JERF3* in tobacco activates downstream gene expression and enhances salt tolerance. Plant Molecular Biology 55: 183-192.

57. Huang ZJ, Zhang ZJ, Zhang XL, Zhang HB, Huang DF, et al. (2004) Tomato *TERF1* modulates ethylene response and enhances osmotic stress tolerance by activating expression of downstream genes. Febs Letters 573: 110-116.

58. Pirrello J, Jaimes-Miranda F, Sanchez-Ballesta MT, Tournier B, Khalil-Ahmad Q, et al. (2006) *Sl-ERF2*, a tomato ethylene response factor involved in ethylene response and seed germination. Plant and Cell Physiology 47: 1195-1205.

59. Chen GP, Hu ZL, Grierson D (2008) Differential regulation of tomato ethylene responsive factor LeERF3b, a putative repressor, and the activator *Pti4* in ripening mutants and in response to environmental stresses. Journal of plant physiology 165: 662-670.

60. Lu CW, Shao Y, Li L, Chen AJ, Xu WQ, et al. (2011) Overexpression of *SlERF1* tomato gene encoding an ERF-type transcription activator enhances salt tolerance. Russian Journal of Plant Physiology 58: 118-125.

61. Pan Y, Seymour GB, Lu CG, Hu ZL, Chen XQ, et al. (2012) An ethylene response factor (*ERF5*) promoting adaptation to drought and salt tolerance in tomato. Plant cell reports 31: 349-360.

62. Lee JM, Joung JG, McQuinn R, Chung MY, Fei ZJ, et al. (2012) Combined transcriptome, genetic diversity and metabolite profiling in tomato fruit reveals that the ethylene response factor *SlERF6* plays an important role in ripening and carotenoid accumulation. Plant Journal 70: 191-204.

63. Kim JG, Stork W, Mudgett MB (2013) Xanthomonas type III effector XopD desumoylates tomato transcription factor *SlERF4* to suppress ethylene responses and promote pathogen growth. Cell Host Microbe 13: 143-154.

64. Li JH, Sima W, Ouyang B, Wang TT, Ziaf K, et al. (2012) Tomato SlDREB gene restricts leaf expansion and internode elongation by downregulating key genes for gibberellin biosynthesis. Journal of Experimental Botany 63: 6407-6420.

65. Park JM, Park CJ, Lee SB, Ham BK, Shin R, et al. (2001) Overexpression of the tobacco *Tsi1* gene encoding an EREBP/AP2-Type transcription factor enhances resistance against pathogen attack and osmotic stress in tobacco. Plant Cell 13: 1035-1046.

66. Fischer U, Droge-Laser W (2004) Overexpression of *NtERF5*, a new member of the tobacco ethylene response transcription factor family enhances resistance to Tobacco mosaic virus. Molecular Plant-Microbe Interactions 17: 1162-1171.

67. Guo ZJ, Chen XJ, Wu XL, Ling JQ, Xu P (2004) Overexpression of the AP2/EREBP transcription factor *OPBP1* enhances disease resistance and salt tolerance in tobacco. Plant Molecular Biology 55: 607-618.

68. Trujillo LE, Sotolongo M, Menendez C, Ochogavia ME, Coll Y, et al. (2008) *SodERF3*, a novel sugarcane ethylene responsive factor (ERF), enhances salt and drought tolerance when overexpressed in tobacco plants. Plant and Cell Physiology 49: 512-525.

69. Liang HX, Lu Y, Liu HX, Wang FD, Xin ZY, et al. (2008) A novel activator-type ERF of *Thinopyrum intermedium*, *TiERF1*, positively regulates defence responses. Journal of Experimental Botany 59: 3111-3120.

70. Li C, Han LB, Zhang XZ (2012) Enhanced Drought Tolerance of Tobacco Overexpressing OjERF Gene Is Associated with Alteration in Proline and Antioxidant Metabolism. Journal of the American Society for Horticultural Science 137: 107-113.

71. Zhai Y, Wang Y, Li YJ, Lei TT, Yan F, et al. (2013) Isolation and molecular characterization of *GmERF7*, a soybean ethylene-response factor that increases salt stress tolerance in tobacco. Gene 513: 174-183.

72. Lai Y, Dang FF, Lin J, Yu L, Shi YL, et al. (2013) Overexpression of a Chinese cabbage *BrERF11* transcription factor enhances disease resistance to *Ralstonia solanacearum* in tobacco. Plant Physiology and Biochemistry 62: 70-78.

73. Zhang HB, Yang YH, Zhang ZJ, Chen J, Wang XC, et al. (2008) Expression of the ethylene response factor gene *TSRF1* enhances abscisic acid responses during seedling development in tobacco. Planta 228: 777-787.

74. Jaglo KR, Kleff S, Amundsen KL, Zhang X, Haake V, et al. (2001) Components of the Arabidopsis C-Repeat/Dehydration-Responsive Element Binding Factor Cold-Response Pathway Are Conserved in *Brassica napus* and Other Plant Species. Plant Physiology 127: 910-917.

75. Boutilier K, Offringa R, Sharma VK, Kieft H, Ouellet T, et al. (2002) Ectopic expression of *BABY BOOM* triggers a conversion from vegetative to embryonic growth. Plant Cell 14: 1737-1749.

76. Savitch LV, Allard G, Seki M, Robert LS, Tinker NA, et al. (2005) The effect of overexpression of two *Brassica* CBF/DREB1-like transcription factors on photosynthetic capacity and freezing tolerance in *Brassica napus*. Plant and Cell Physiology 46: 1525-1539.

77. El Ouakfaoui S, Schnell J, Abdeen A, Colville A, Labbe H, et al. (2010) Control of somatic embryogenesis and embryo development by AP2 transcription factors. Plant Molecular Biology 74: 313-326.

78. Xiong AS, Jiang HH, Zhuang J, Peng RH, Jin XF, et al. (2013) Expression and Function of a Modified AP2/ERF Transcription Factor from *Brassica napus* Enhances Cold Tolerance in Transgenic *Arabidopsis*. Molecular Biotechnology 53: 198-206.
